# The Active Components of Fuzheng Huayu Formula and Their Potential Mechanism of Action in Inhibiting the Hepatic Stellate Cells Viability – A Network Pharmacology and Transcriptomics Approach

**DOI:** 10.3389/fphar.2018.00525

**Published:** 2018-05-24

**Authors:** Xinrui Xing, Si Chen, Ling Li, Yan Cao, Langdong Chen, Xiaobo Wang, Zhenyu Zhu

**Affiliations:** ^1^School of Pharmacy, Second Military Medical University, Shanghai, China; ^2^Postdoctoral Research Workstation, 210th Hospital of the Chinese People’s Liberation Army, Dalian, China

**Keywords:** Fuzheng Huayu formula, network pharmacology, transcriptomics, liver fibrosis, pharmacological mechanism

## Abstract

**Purpose:** This study aimed to identify the active components of Fuzheng Huayu (FZHY) formula and the mechanism by which they inhibit the viability of hepatic stellate cells (HSCs) by a combination of network pharmacology and transcriptomics.

**Methods:** The active components of FZHY formula were screened out by text mining. Similarity match and molecular docking were used to predict the target proteins of these compounds. We then searched the STRING database to analyze the key enriched processes, pathways and related diseases of these target proteins. The relevant networks were constructed by Cytoscape. A network analysis method was established by integrating data from above network pharmacology with known transcriptomics analysis of quiescent HSCs-activated HSCs to identify the most possible targets of the active components in FZHY formula. A cell-based assay (LX-2 and T6 cells) and surface plasmon resonance (SPR) analysis were used to validate the most possible active component-target protein interactions (CTPIs).

**Results:** 40 active ingredients in FZHY formula and their 79 potential target proteins were identified by network pharmacology approach. Further network analysis reduced the 79 potential target proteins to 31, which were considered more likely to be the target proteins of the active components in FZHY formula. In addition, further enrichment analysis of 31 target proteins indicated that the HIF-1, PI3K-Akt, FoxO, and chemokine signaling pathways may be the primary pathways regulated by FZHY formula in inhibiting the HSCs viability for the treatment of liver fibrosis. Of the 31 target proteins, peroxisome proliferator activator receptor gamma (PPARG) was selected for validation by experiments at the cellular and molecular level. The results demonstrated that schisandrin B, salvianolic acid A and kaempferol could directly bind to PPARG, decreasing the viability of HSCs (T6 cells and LX-2 cells) and exerting anti-fibrosis effects.

**Conclusion:** The active ingredients of FZHY formula were successfully identified and the mechanisms by which they inhibit HSC viability determined, using network pharmacology and transcriptomics. This work is expected to benefit the clinical application of this formula.

## Introduction

Liver fibrosis is a protective mechanism of organ integrity with the accumulation of extracellular matrix and collagen proteins, which occurs as a result of large-scale apoptosis and necrosis (Fagone et al., 2016). It is mainly associated with chronic viral hepatitis type B or viral hepatitis type C infection, biliary diseases, alcoholic steatohepatitis, and nonalcoholic steatohepatitis (Friedman, 2008). In 2004, the leading cause of liver fibrosis was hepatitis type B in China, and viral hepatitis type C, alcoholic steatohepatitis and nonalcoholic steatohepatitis in United States and most European countries (Afdhal, 2004; Lavanchy, 2004; McMahon, 2004). Without timely and effective treatment, liver fibrosis will gradually evolve into cirrhosis and even liver carcinoma, leading to death.

A growing body of evidence has shown that liver fibrosis is a reversible process (Hernandez-Gea and Friedman, 2011; Wang et al., 2015; Zhang C.Y. et al., 2016). Since the activation of hepatic stellate cells (HSCs) is the primary process of hepatic fibrosis (Schuppan and Kim, 2013), inhibiting the activation of HSCs should be an effective treatment (Liu et al., 2015). Currently, most researchers developed new drugs for liver fibrosis based on the “one drug for one target for one disease” assumption (Mao and Fan, 2015; Koyama et al., 2016; Wang P. et al., 2016). For example, Sorafenib can inhibit the proliferation of HSCs through targeting tyrosine kinase (Mejias et al., 2009); and Rimonabant, a cannabinoid receptor type 1 antagonist, can promote apoptosis of activated HSCs (Dai et al., 2014). But the clinical utility of both drugs is limited by their off-target effects to cause severe adverse reactions. And network pharmacology studies concluded liver fibrosis to be of a complicated etiology, unamenable to effective treatment by intervention at a single node (Liu et al., 2006). Accordingly, safer and more effective treatment strategies for hepatic fibrosis are urgently needed.

Traditional Chinese Medicine (TCM) formulae have been widely used in clinical practice for thousands of years due to their efficacy and lack of serious side effects, and are an indispensable part of the current medical service system (Zhang Y. et al., 2016). One characteristic of TCM formulae is that they generally comprise multiple ingredients, which operate in synergy and on multiple disease targets (Li and Zhang, 2013). FZHY recipe, a classic TCM formula originally called Ganping capsule and 319 recipe, has been approved as an anti-fibrotic medicine by the State Food and Drug Administration. And it recently completed phase II clinical trials in the United States. This formula consists of six different herbs: *Salviae miltiorrhizae* Radix et Rhizoma (derived from *Salvia miltiorrhiza* Bge., Danshen; *S. miltiorrhiza*), Semen Persicae [derived from *Prunus persica* (L.) Batsch, Tao Ren; *S. Persicae*], Cordyceps sinensis [derived from *Cordyceps sinensis* (BerK.) Sacc., Dong Chong Xia Cao; *C. sinensis*], Gynostemma pentaphyllum [derived from *Gynostemma pentaphyllum* (Thunb.) Makino, Jiao Gu Lan; *G. pentaphyllum*], Schisandra chinensis Fructus [derived from *Schisandra chinensis* (Turcz.) Baill., Wu Wei Zi; *S. chinensis*] and Pollen Pini (derived from *Pinus massoniana* Lamb., Song Hua Fen; *P. Pini*). It has been shown to significantly improve liver function and clinical symptoms, decrease collagen synthesis, promote degradation of extracellular matrix, reverse hepatic fibrosis, and reduce mental stress in patients with chronic hepatitis B and liver cirrhosis (Jia et al., 2012). Furthermore, the FZHY formula can reduce oxidative stress by down-regulating cytochrome P450 2E1 and tumor necrosis factor receptor type I (Jia et al., 2012) expression. Also, several researchers reported the antifibrotic capability of the FZHY recipe in the treatment of liver fibrosis through inhibition of HSC activation (Tao et al., 2014; Pan et al., 2015; Li et al., 2016). However, neither the active components of the FZHY formula nor their therapeutic target proteins in inhibiting HSCs viability are clear.

In the emerging paradigm of network pharmacology, the concepts of drug discovery and drug development are moved from a “one target-one drug” mode to a “multi-target-multi-component” mode (Li, 2011; Li et al., 2011; Xu et al., 2014). This latter mode in network pharmacology coincides well with the integrity and systemic nature of TCM formulae, which could solve above problems of the FZHY formula. Previous studies in our laboratory confirmed the utility of network pharmacology to clarify the synergistic molecular mechanisms of *Sini* decoction and *Ku Shen* (Yang et al., 2013; Chen et al., 2014; Wang S.Q. et al., 2016). However, network pharmacology approaches routinely yield hundreds of potential target proteins for the active components in TCM formula (Liu et al., 1999; Jia et al., 2012; Wang et al., 2012), and selection of the target proteins most suitable for validation is difficult. As a combination of approaches is thought most likely to bear fruit, we hypothesized that the combination of network pharmacology and transcriptomics could increase the accuracy of target identification of network pharmacology.

**FIGURE 1:**
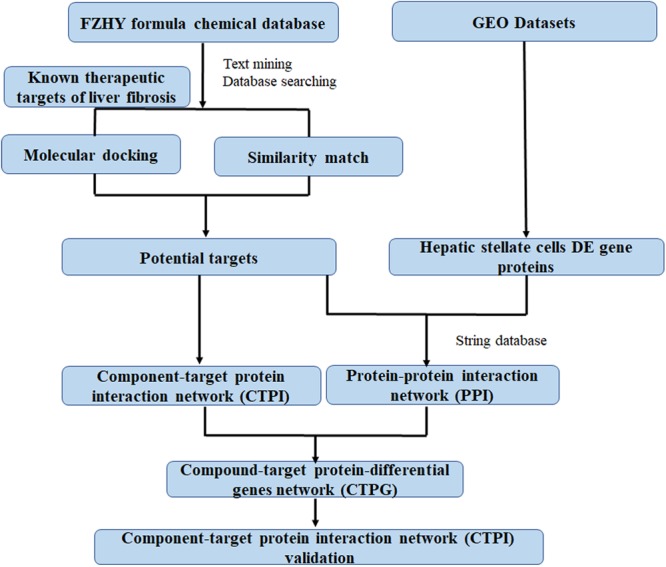
The flowchart of network analysis approach.

In this study, network pharmacology and transcriptomics were used to identify the active ingredients in the FZHY formula, and the mechanisms by which they diminished HSCs viability. Subsequently, these predictions were verified in a series of experiments. A detailed flowchart is depicted in **Figure 1**. This is the first study to contemplate the mechanism of action of FZHY formula in the inhibition of HSCs viability for the treatment of liver fibrosis by the methods of network pharmacology and transcriptomics.

## Materials and Methods

### Database Construction

Information (structure, canonical name, and component identification number) pertaining to the compounds present in FZHY formula was obtained from the TCMs Integrated Database^1^ (Xue et al., 2013), the TCM Database@Taiwan^2^ (Chen, 2011), and the Chemistry Database^3^. The active compounds of all above herbs were found out by text mining from the National Center for Biotechnology Information PubMed database^4^. The search bar was comprised of a compound name in FZHY formula and liver fibrosis or hepatic fibrosis.

Candidate target proteins relating to liver fibrosis were obtained by searches of the PubMed database. The most appropriate crystallographic structures of each target protein were then downloaded from the UniProt Database^5^ and RCSB Protein Data Bank^6^.

### Target Prediction

Potential target proteins of the FZHY formula were identified using two methods. Firstly, since approved drugs of a similar structure may have common target proteins and similar curative effects, we used the drug similarity search tool in ChEMBL^7^ to identify compounds similar to the active components of the FZHY formula. Only compounds with a high similarity score (≥0.95) as compared with the structures of active components in FZHY formula were picked out, in order to obtain more accurate results. The therapeutic target proteins of these similar compounds were also collected in ChEMBL; those implicated in liver fibrosis were hypothesized to be targeted by the related active component of the FZHY formula. Secondly, molecular docking, which is very useful in rational drug design, can be used not only to predict the binding sites and pose(s) of drug candidates with their target proteins, but also to evaluate their binding affinities (Kitchen et al., 2004). We used libdock in Discovery Studio 3.0^8^ to predict possible relationships between the target proteins of liver fibrosis and the active components of FZHY. The co-crystallized ligand binding with the target protein was regarded as a positive control. The dock scores of the positive control with corresponding proteins were regarded as cutoff point. If the dock score is higher than the cutoff point, the protein will be recognized as a potential target protein of the compounds. Based on these results, an active component-target protein interaction (CTPI) network can be constructed and displayed using Cytoscape 3.5.1 (Smoot et al., 2011). A component and a related potential target protein are linked with an edge; and components or target proteins are represented by nodes.

### Gene Expression Profiles

Gene expression microarray data (GSE68001) of primary human quiescent HSCs and *in vitro* activated HSCs were obtained from the National Center for Biotechnology Information Gene Expression Omnibus^9^, a public functional genomics data repository.

### Network Construction and Analysis

Based on the acquired identities of the potential target proteins and differential genes relating to the activation of HSCs, a protein-protein interaction (PPI) network was built by importing the gene names of above proteins and genes to the public database STRING (version 10.5^10^). The minimum required interaction score was set at 0.9, to improve the accuracy of the results. Cytoscape 3.5.1 (Smoot et al., 2011) were used as a tool to visualize the PPI network. Subsequently, PPI combined with CTPI was used to build a new compound-target protein-differential genes (CTPG) network. This network analysis process can identify target proteins which can connect compounds in FZHY formula with differential genes. To facilitate scientific interpretation of identified potential targets, a STRING database was used to perform several analyses such as gene ontology (GO) enrichment analysis and pathway enrichment analysis.

### Quality Control of FZHY Formula

The FZHY formula was purchased from Yifeng Pharmacy (Shanghai, China), and extracted by sonication in methanol for 60 min. UPLC-Q-TOF/MS was used to identify the active components of FZHY. Mass spectra was acquired in both negative and positive modes, and non-target compound identification was further conducted based on obtained fingerprints. Formulae were proposed based on the mass spectra and other rules, such as the general rule of the number of nitrogen atoms, double bond equivalent (DBE) index and ‘show isotopic’ function. And the compound was finally confirmed by the comparison with the authentic compound.

### Materials and Reagents

Salvianolic acid B, dihydrotanshinone I, salvianolic acid A, tanshinone II-A, amygdalin, adenosine, cordycepin, schizandrin, schisandrin B, schisantherin A, gypenoside XLIX and kaempferol were purchased from EFEBIO (Shanghai, China^11^), and their structural information is shown in Supplementary Figure S1. 15-deoxy-Δ12,14-prostaglandin J2 (15d-PGJ2) was used as a positive control (Jin et al., 2016), and obtained from Abcam (Cambridge, United Kingdom). The structures of the above chemicals were unambiguously identified by ^1^H NMR and MS spectra, and their purity was demonstrated to be 98% by HPLC-UV. Cell Counting Kit-8 (CCK8) detection kit was obtained from Beyotime (Shanghai, China). LX-2 cells and T6 cells were purchased from Cell Resource Center of Fudan IBS (Shanghai, China). These cells were incubated in Dulbecco minimal essential medium (Sigma, United States) with 10% fetal bovine serum (GIBCO, United States) and penicillin, streptomycin (GIBCO, United States) under a humidified atmosphere with 5% CO_2_ at 37°C. The medium was renewed every 2 days.

### Cell Proliferation Assay

A total of 5 × 10^3^ cells were planted in 96 well plates and cultivated for 24 h. Then, cells were treated with various concentrations of compounds (6.25, 12.5, 25, 50, 100, 200 μM) and 15d-PGJ2 (100 μM) for 24 h. Cell viability was tested by CCK-8; the absorbance was directly detected using a Bio-Rad microplate reader (Synergy^TM^ 4, BioTek, United States) at 450 nm. All experiments were repeated three times.

### Surface Plasmon Resonance (SPR) Analysis

Surface plasmon resonance (SPR) analyses were undertaken at 25°C on a BIA core T200 instrument (GE Healthcare, Little Chalfont, Buckinghamshire, United Kingdom), using a phosphate buffered solution with 5% dimethyl sulfoxide as running buffer, with a constant flow rate of 30 ml/min. Peroxisome proliferator-activated receptor gamma (PPARG, Proteintech) protein was immobilized on CM5 chips with levels of 2446.2 by applying 1-(3-dimethylaminopropyl)-3-ethylcarbodiimide/N-hydroxy succinimide cross-linking reaction. The detection was performed according to the protocol provided by GE Healthcare. Gradient concentrations of components (0.5–256 μM) were dissolved in the running buffer and then injected into the channel for 60 s followed by disassociation for 120s. BIA evaluation 3.0 software (BIAcore) was used to analyze the data by a 1:1 binding model.

## Results and Discussion

### Active Components and Their Potential Target Proteins of FZHY Formula for the Treatment of Liver Fibrosis

Forty active compounds of FZHY formula were retrieved from the PubMed database (**Table 1**). A molecular docking and similarity match were used to identify potential target proteins of active compounds in the FZHY formula. A total of 79 potential target proteins (Supplementary Table S1) were obtained, of which 45 potential target proteins came from similarity match, and 39 from docking with targets of liver fibrosis (Supplementary Table S2). Interestingly, the PPARG, STAT3, PGFRB, KS6B1, MK08 target proteins were found by both two approaches. Specifically, there were 14 active compounds in *S. miltiorrhiza* targeting 43 potential proteins; 12 active compounds in *C. sinensis* targeting 32 potential proteins; 6 active compounds in *G. pentaphyllum* targeting 31 potential proteins; one active compound in *P. Pini* targeting 35 potential proteins; five active compounds in *S. Persicae* targeting 31 potential proteins; and five active compounds in *S. chinensis* targeting 22 potential proteins.

**Table 1:** Active components identified by in six herbs.

Herbs	Number	Components
*S. miltiorrhiza*	14	Salvianolic acid A, baicalin, dihydrotanshinone I, rosmarinic acid, ursolic acid, danshensu, ferulic acid, magnesium lithospermate B, protocatechuic aldehyde, tanshinol, rutin, tanshinone II-A, salvianolic acid B, β-sitosterol
*C. sinensis*	12	Histidine, ergosterol, valine, adenosine, ascorbic acid, vitamin B12, vitamin A, nicotinic acid, glycine, cordycepin, linoleic acid, β-sitosterol
*S. chinensis*	8	β-caryophyllene, β-elemene, schizandrin, vitamin k1, schisandrol B, schisantherin A, schisandrin B, β-sitosterol
*G. pentaphyllum*	7	Ginsenoside-rb1, rutin, ginsenoside-rb2, gypenoside XLIX, gipsoside, gypenoside A, β-sitosterol
*S. Persicae*	4	Chlorogenic acid, amygdalin, (+)-catechin, β-sitosterol
*P. Pini*	1	Kaempferol

### Compound-Target Protein Network Construction and Analysis

**FIGURE 2:**
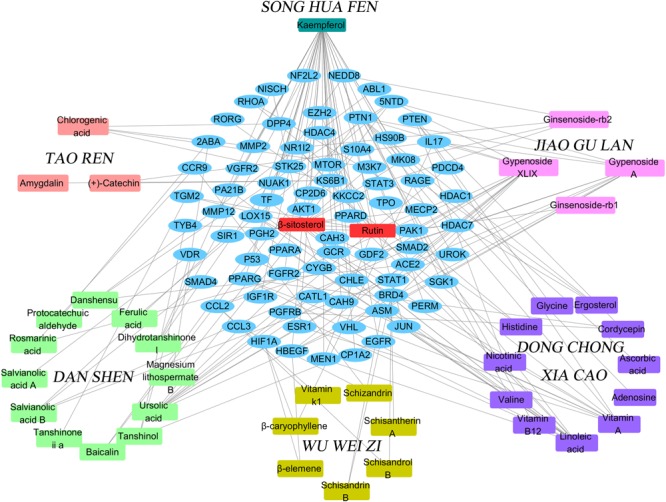
The Component-Target protein network. The rectangles represent the 45 candidate compounds in Fuzheng Huayu (FZHY), the cyan, red, green, yellow, purple, pink rectangles refers to active compounds in *Pinus massoniana* Lamb. (Song Hua Fen), *Prunus persica* (L.) Batsch (Tao Ren), *Salvia miltiorrhiza* Bunge (Dan Shen), *Schisandra chinensis* (Turcz.) Baill. (Wu Wei Zi), *Cordyceps sinensis* (BerK.) Sacc. (Dong Chong Xia Cao), *Gynostemma pentaphyllum* (Thunb.) Makino (Jiao Gu Lan), respectively. The red rectangles in the center of network represent the common components of several herbs. The blue circles represent the gene names of target proteins of the six herbs found by text mining and molecular docking.

We used the above data to construct a CPTI (**Figure 2**), which contains 119 nodes (40 active compounds and 79 potential targets) and 172 edges. In this network, the rectangles and ellipses represent the active compounds and their target proteins, respectively. There are two red rectangles in the middle of the blue ellipse, which represent the common components of several herbs in FZHY formula. Specifically, β-sitosterol was present in *S. miltiorrhiza, S. Persicae, C. sinensis, G. pentaphyllum*, and *S. chinensis*; and rutin was an ingredient of both *S. miltiorrhiza* and *S. chinensis* β-sitosterol and rutin may be the key active compounds of FZHY formula. As shown in **Figure 2**, there are more active compounds in *S. miltiorrhiza* than in the other five herbs, and the active compounds in *S. miltiorrhiza* targeted the largest number of proteins compared with the active compounds in other five herbs. Thus, we infer that *S. miltiorrhiza* is the principal ingredient of FZHY, which is consistent with previous work (Zhao C.Q. et al., 2006; Yang et al., 2015).

**FIGURE 3:**
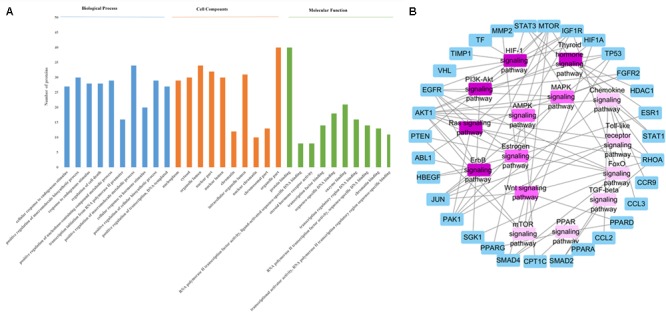
**(A)** The enrichment analysis in biological processes, cellular components and molecular functions of 79 identified target proteins by STRING database. **(B)** The Target protein-Pathway network. Blue nodes refer to target proteins. The pink nodes represent pathways, and the color is consistent with pathways related target protein numbers, the deeper the pink, the more number of proteins.

STRING (version 10.5, see foot note text 10) is a database which aims to collect and integrate all functional interactions between expressed proteins by consolidating known and predicted protein-protein association data for a large number of organisms. This platform can also be used to conduct functional enrichments of user inputs. In this study, we performed the gene ontology enrichment analysis and pathway enrichment analysis of potential target proteins with the functions in STRING. The meaningful pathways (Supplementary Table S4), biological processes (Supplementary Table S3), cellular components (Supplementary Table S5) and molecular functions (Supplementary Table S6) were selected with a *p*-value < 0.05. **Figure 3A** depicts the enriched molecular functions of the target protein, which are mainly associated with binding and steroid hormone receptor activity. The binding activities are primarily connected with protein binding and enzyme binding. As to the cellular components’ distribution, the target proteins were mainly distributed in nuclear part, organelle part and organelle lumen (**Figure 3A**). The main biological processes of the target proteins are also summarized in **Figure 3A**, which shows that the active ingredients in FZHY formula could exert an anti-liver fibrosis effect through cellular response to endogenous stimulus, positive regulation of macromolecule biosynthetic process, response to endogenous stimulus, regulation of cell death, positive regulation of nucleobase-containing compound metabolic process, transcription initiation from RNA polymerase II promoter, positive regulation of macromolecule metabolic process, cellular response to hormone stimulus, positive regulation of cellular biosynthetic process, and positive regulation of transcription. Among these processes, FZHY formula had been reported to cure liver fibrosis by positive regulation of macromolecule metabolic process (Gao et al., 2016) and positive regulation of macromolecule biosynthetic process (Liu et al., 2006; Cheng et al., 2013). In addition, cellular responses to endogenous stimulus (Huang et al., 2016) and regulation of cell death (Zhong et al., 2017) have been reported to be closely related to liver fibrosis, further experiments are needed to validate the interaction between FZHY formula and these two biological processes.

In order to identify the significant pathways that the target proteins are involved in, we made a pathway enrichment analysis of target proteins by STRING database (**Figure 3B**). Logically, the pathway that contains more target proteins is more important than the pathway that contains fewer target proteins. Hence, a target protein-pathway network (**Figure 3B**) was constructed to indicate the most important pathways. The results showed that the FZHY formula exerted its protective effects against liver fibrosis primarily by regulating 15 pathways (**Figure 3B**), of which the TGF-beta signaling pathway has been reported (Wang et al., 2012). In addition, although lots of references indicated that liver fibrosis was closely related to the other 14 pathways, such as hypoxia inducible factor-1 (HIF-1) signaling pathway (Zhao et al., 2014), the forkhead box O (FoxO) signaling pathway (Wu et al., 2015), the ErbB signaling pathway (Scheving et al., 2016), the Chemokine signaling pathway (Marra and Tacke, 2014), the thyroid hormone signaling pathway (Bai et al., 2014), the estrogen signaling pathway (Deng and He, 2007), the Ras signaling pathway (Abbas et al., 2011), the phosphatidylinositol-3-kinase (PI3K-Akt) signaling pathway (Zhang D.Q. et al., 2016), the adenosine 5′-monophosphate (AMP)-activated protein kinase (AMPK) signaling pathway (Bai et al., 2014), the mammalian target of rapamycin (mTOR) signaling pathway (Li et al., 2015), the PPAR signaling pathway (Jiang et al., 2015), the mitogen-activated protein kinase (MAPK) signaling pathway (Park et al., 2015), the toll-like receptor signaling pathway (Aoyama et al., 2010), and the Wnt signaling pathway (Miao et al., 2013); further experiments are needed to validate these findings.

Taken together, FZHY formula may exert an anti-liver fibrosis effect primarily by regulating 10 biological processes and 15 pathways. The multiple active components in FZHY formula target multiple proteins in the biological network to regulate and restore the network equilibrium, thereby mitigating the development of liver fibrosis.

### Construction and Analysis of Target Protein-Disease Network

**FIGURE 4:**
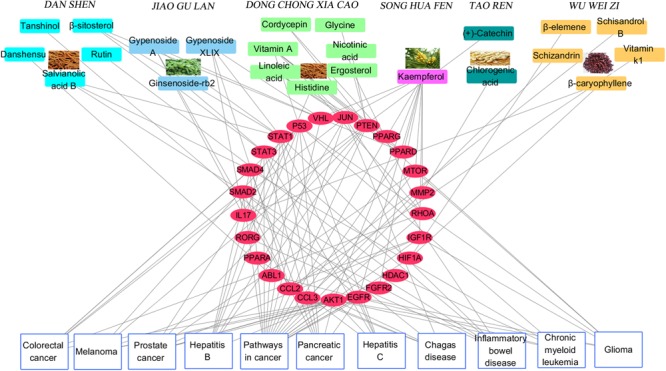
Component-Target protein-Disease network. The Top rectangles in the network represent active components of six herbs in FZHY, red ellipse nodes in the Middle of the network denote target proteins of active compounds, the Bottom rectangles refer to diseases correlated with above target proteins.

**Figure 4** depicts the classification of the target-related diseases, and indicates that FZHY formula can show a protective effect against pancreatic cancer, prostate cancer, colorectal cancer, glioma, inflammatory bowel disease, hepatitis B, hepatitis C, melanoma, chronic myeloid leukemia, and chagas disease. The utility of FZHY for the treatment of hepatitis B (Tang et al., 2012), hepatitis C (Zhang B. et al., 2014), liver cirrhosis (Chen et al., 2016), and steatohepatitis (Jia et al., 2012) has been previously reported; however, we were unable to find reports pertaining to its efficacy against cancer, inflammatory bowel disease, chronic myeloid leukemia and chagas disease.

### Compound-Target Protein-Differential Gene Protein Network Analysis

**FIGURE 5:**
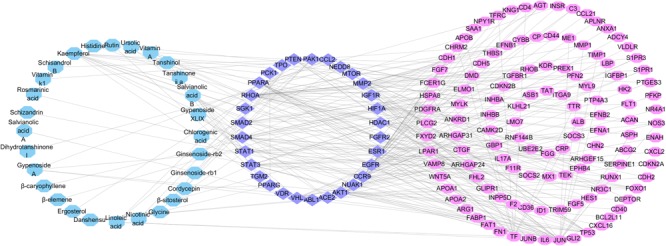
Compound-Target protein-Differential gene protein network. This network was built by linking 28 active components (blue rectangles) in FZHY formula, 31 target proteins (purple ellipses) and 122 different gene proteins (pink ellipses).

In order to improve the accuracy of target identification, a compound-target protein-differential gene protein network (CTPG) was established through the integration of CTPI and PPI (**Figure 5**). This network consists of chemical components, target proteins and differential gene proteins, including 179 nodes and 31862 edges. The components acting on target proteins cause the up- or down-regulation of related differential gene proteins. The interactions between target proteins and differential gene proteins were obtained from the STRING database with high confidence (>0.9). The active components were considered as the initial nodes, and the target proteins and differential gene proteins were as the terminal nodes. **Figure 5** depicts 28 components interacted with 31 target proteins and 122 differential gene proteins. The 31 target proteins in this network are more likely to be the true targets of the active components in FZHY compared to the 79 target proteins identified from network pharmacology. This deduction has been proved by reference–briefly, a total of 79 potential target proteins were identified by network pharmacology, but only 40 of these were validated to be related to liver fibrosis. Of the 31 target proteins identified by network analysis, 25 (Supplementary Table S8) are in agreement with existing research results. Target proteins related to liver fibrosis validated by references are summarized in Supplementary Table S7.

We randomly selected signal transducer and activator of transcription 1 (STAT1) and PPARG identified by network analysis as examples to explain the potential synergistic mechanism of the active components in FZHY for alleviating liver fibrosis through inhibition of HSCs activation (**Figure 5**). In addition, heparin-binding epidermal growth factor-like growth factor (HBEGF) was also selected representatively from the other 15 liver fibrosis related target proteins predicted only by network pharmacology.

Previous researches showed that the activation of STAT1 could attenuate liver fibrosis through inhibiting HSCs activation and proliferation (Thomas et al., 2004; Jeong et al., 2006), and promoting apoptosis by inhibiting anti-apoptotic genes or inducing pro-apoptotic genes in different tissues (Stephanou and Latchman, 2003). As STAT1 was identified as a target of the active components in FZHY formula (**Figure 5**), one possible explanation for the liver fibrosis-related medicinal properties of FZHY formula is that it inhibits the activation and proliferation of HSCs through activation of STAT1.

Peroxisome proliferator activator receptor gamma was proposed as a crucial factor for the inhibition of HSCs activation (Hazra et al., 2004; Tsukamoto, 2005; Yang et al., 2006). The level of PPARG was higher in quiescent HSCs, compared to activated HSCs; and its abundance, expression, and transcriptional activity were all suppressed during HSCs activation *in vivo* and *in vitro* (Galli et al., 2000; Miyahara et al., 2000; She et al., 2005). That up-regulation of PPARG expression could suppress the HSCs activation (Zhai et al., 2015), a pivotal event in the pathology of liver fibrosis (Liu et al., 2017), was also shown. Thus, increasing PPARG expression levels in HSCs should be an effective means of liver fibrosis prevention (Galli et al., 2000, 2002; Xu et al., 2003). Furthermore, some PPARG agonists, such as troglitazone, thiazolidinediones, saroglitazar, KR62776, GW7845, 6-octadecynoic acid, and 15d-PGJ2, have been proven to attenuate liver fibrosis by cell-based or animal experiments (Marra et al., 2000; Galli et al., 2002; Zhao C. et al., 2006; Bae et al., 2010; Ohtera et al., 2013; Jain et al., 2017). PPARG agonists can therefore be concluded to be effective treatments for liver fibrosis by inhibiting the HSC activation; and PPARG is a promising therapeutic target for antifibrotic chemotherapy (Zhang F. et al., 2013). Thus, the active components in FZHY formula may inhibit HSCs viability by targeting PPARG. Interestingly, one component of FZHY formula, ergosterol, was shown to cure liver fibrosis by upregulation of the expression of PPARG in HSC-T6 cells (Tai et al., 2016). In addition, rosmarinic acid and baicalin (both also present in FZHY formula) were identified to impart an antifibrotic effect by epigenetic expression repress of PPARG (Yang et al., 2012; Tai et al., 2016).

Heparin-binding epidermal growth factor, which was identified by network pharmacology and not of network analysis, has also been reported to serve as a fibrosis inducer (Guo et al., 2017). Also, other studies indicated that HBEGF inhibitor could cure liver fibrosis by efficiently inhibiting HSCs activation (Zhang D. et al., 2014). Thus, we inferred that the active components in FZHY formula may inhibit HSCs activation to cure liver fibrosis through obstruction of HBEGF. This deduction not only demonstrates the reliability of the network pharmacology method, but also exemplifies the limitation of the network analysis approach, the precision of which needs improvement.

In summary, the active components of FZHY counter liver fibrosis by simultaneously targeting STAT1, PPARG and HBEGF, to prevent HSCs activation, proliferation and migration.

### Experimental Target Validation

Peroxisome proliferator activator receptor gamma was selected for experimental validation, for three reasons. Firstly, PPARG was identified by both text mining and molecular docking (**Figure 2**). Secondly, PPARG was present in the compound-target protein-differential gene protein network. Thirdly, numerous references have implicated the important role of PPARG in anti-fibrosis (Iwaisako et al., 2012; Nan et al., 2013; Zhao et al., 2013; Liu et al., 2014; Tomita et al., 2014). Moreover, three active components in FZHY formula were verified to inhibit HSCs activation by targeting of PPARG (Yang et al., 2012; Tai et al., 2016). Thus, we inferred that PPARG could be the primary target of several active components in FZHY formula.

Salvianolic acid B, dihydrotanshinone I, salvianolic acid A, tanshinone II-A, amygdalin, adenosine, cordycepin, schizandrin, schisandrin B, schisantherin A, gypenoside XLIX and kaempferol as representative components of each herb in FZHY formula were chosen to test their interaction with PPARG, having been shown by UPLC-Q-TOF/MS analysis to be present in FZHY formula (Supplementary Figures S2–S13). And active compounds, such as kaempferol, schisantherin A, schisandrin B, tanshinone II-A, salvianolic acid A, dihydrotanshinone I, salvianolic acid B, and cordycepin, were verified to be stable in DMSO under a humidified atmosphere with 5% CO_2_ at 37°C for 24 h by HPLC-UV, related HPLC chromatograms can be seen in Supplementary Figures S14–S21.

#### Active Components of FZHY Formula Inhibit the Viability of T6 and LX-2 Cells

**FIGURE 6:**
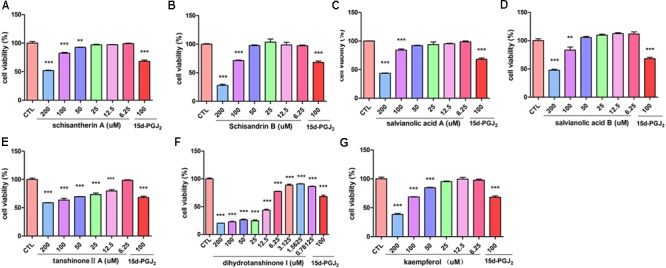
Active components in FZHY inhibited human HSC-LX-2 activation. LX-2 cells were treated for 24 h with indicated concentrations of **(A)** schisantherin A, **(B)** schisandrin B, **(C)** salvianolic acid A, **(D)** salvianolic acid B, **(E)** tanshinone II-A, **(F)** dihydrotanshinone I, **(G)** kaempferol. CCK8 assay was used to measure the cell viability. Data were obtained from three independent experiments performed in triplicate and presented as means (±SD). ^∗^*P* < 0.05, ^∗∗^*P* < 0.01, ^∗∗∗^*P* < 0.001 versus the control (CTL).

**FIGURE 7:**
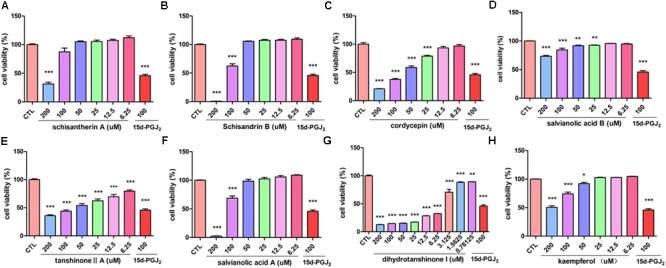
Active components of FZHY formula inhibited the activation of rat HSC-T6 cells. T6 cells were treated for 24 h in the presence of indicated concentrations of **(A)** schisantherin A, **(B)** schisandrin B, **(C)** cordycepin, **(D)** salvianolic acid B, **(E)** tanshinone II-A, **(F)** salvianolic acid A, **(G)** dihydrotanshinone I, **(H)** kaempferol, and the cell viability was measured with CCK8 assay. Data were obtained from three independent experiments performed in triplicate and presented as means (±SD). ^∗^*P* < 0.05, ^∗∗^*P* < 0.01, ^∗∗∗^*P* < 0.001, compared with the control (CTL).

The inhibitory effect of potential active components of FZHY formula against LX-2 cells and T6 cells were tested; 15d-PGJ2 was selected as a positive control (Jin et al., 2016). Salvianolic acid B, dihydrotanshinone I, salvianolic acid A, tanshinone II-A, cordycepin, schisandrin B, schisantherin A and kaempferol were all found able to inhibit the viability of LX-2 cells (**Figure 6**); IC_50_ values are summarized in **Table 2**. Salvianolic acid B, dihydrotanshinone I, salvianolic acid A, tanshinone II-A, schisandrin B, schisantherin A and kaempferol could all inhibit the viability of T6 cells (**Figure 7**); IC_50_ values are summarized in **Table 2**. Of note, dihydrotanshinone I (100 μM) was a more potent inhibitor of both LX-2 and T6 cell lines than 15d-PGJ2 (100 μM).

**Table 2:** IC_50_ values of active components with LX-2 and T6 cell lines.

Component	IC_50_ (μM) LX-2	IC_50_ (μM) T6
Kaempferol	153.7	196.1
Schisantherin A	>200	164.6
Schisandrin B	141.3	102.8
Tanshinone II-A	>200	65.8
Salvianolic acid A	129.8	113.3
Dihydrotanshinone I	14.4	5.6
Salvianolic acid B	191.3	>200
Cordycepin	none	68.6

#### Active Ingredients in FZHY Formula Directly Binds to PPARG

**FIGURE 8:**
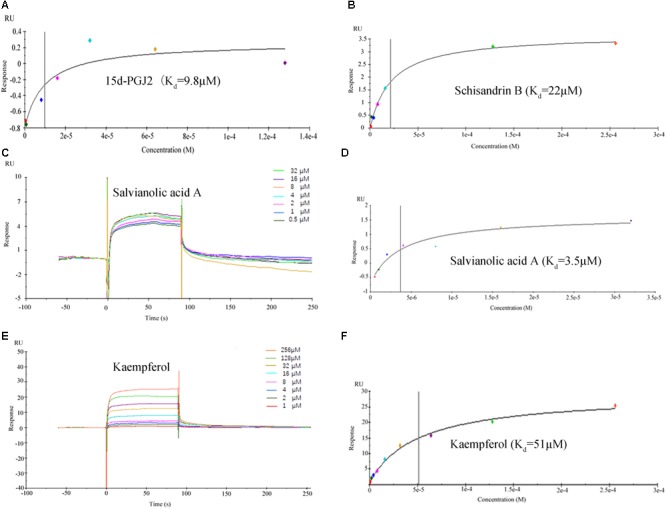
Surface plasmon resonance (SPR) analysis showed that 15d-PGJ2, kaempferol, salvianolic acid A and schisandrin B could directly bound to peroxisome proliferator activator receptor gamma (PPARG). **(A,B,D,F)** The fitting curves and *K*_d_ value of 15d-PGJ2, schisandrin B, salvianolic acid A, kaempferol. **(C,E)** The sensorgrams of salvianolic acid A and kaempferol, which indicate the direct binding of salvianolic acid A and kaempferol to PPARG immobilized on a sensor chip. The kinetic measurements were performed in triplicate using a set of serial dilutions as shown. Data in **(A–F)** were representatives of three independent experiments.

The binding affinities (*K*_d_) of the above 10 active compounds for PPARG were measured in a SPR-based binding assay; three of them exhibited moderate binding activity with PPARG (**Figure 8**). The previously identified agonist 15d-PGJ2 was used as a positive control, and a *K*_d_ value for PPARG of 9.8 μM was calculated, similar to the values reported in the literature. Salvianolic acid A was found to be the most potent agonist tested, with a *K*_d_ value of 3.5 μM (**Figure 8**). Kaempferol and schisandrin B (1–256 μM) also brought about a concentration-dependent resonance change when flowing through the sensor chip coated with PPARG, indicating the direct binding of kaempferol and schisandrin B to PPARG (**Figure 8**). The equilibrium dissociation constant (*K*_d_) was calculated to be 51 and 22 μM. These results are in consistent with the cell viability results, which demonstrated that kaempferol, salvianolic acid A and schisandrin B could bind with PPARG to exert their effects against T6 and LX-2 cells.

## Conclusion

A novel network analysis method for the analysis of FZHY formula has been developed, and used to elucidate the mechanistic basis for the anti-liver fibrosis effects of FZHY formula. Text-mining was first used to identify 40 potential anti-liver fibrosis components in FZHY. Then, 79 potential targets of these components were identified by molecular docking and similarity match. To improve the accuracy of target identification, we combined above network pharmacology results with known transcriptomics results, which reduced the number of possible active components of FZHY formula from 40 to 28, and the number of possible target proteins from 79 to 31. Biological process and pathway enrichment analyses of these target proteins demonstrated that FZHY formula could exert its observed anti-liver fibrotic effect by regulating 10 biological processes and 15 pathways. PPARG was selected to validate the compound-target protein network identified by network analysis. The results indicated that schisandrin B, salvianolic acid A, and kaempferol could directly bind to PPARG, decreasing HSCs viability (T6 cells and LX-2 cells). These findings are expected to inform future research into liver fibrosis treatments; and the network analysis method used is expected to be amenable to the study of other TCM formula.

## Author Contributions

XX is the first author and performed all the experiments and drafted the manuscript. SC conceived the idea, designed the experimental plan and revised the whole manuscript. LL helped the first author, prepared the figures and materials. YC and LC provided technical assistance of SPR. ZZ and XW contributed toward study design, experimental setup, results supervision, and manuscript correction.

## Conflict of Interest Statement

The authors declare that the research was conducted in the absence of any commercial or financial relationships that could be construed as a potential conflict of interest.

## Footnotes

1 www.megabionet.org/tcmid

2 tcm.cmu.edu.tw

3 http://www.organchem.csdb.cn

4 https://www.ncbi.nlm.nih.gov/pubmed/

5 http://www.uniprot.org/

6 http://www.pdb.org/

7 https://www.ebi.ac.uk/chembl/

8 http://www.accelrys.com

9 https://www.ncbi.nlm.nih.gov/gds/

10 https://string-db.org/

11 http://www.efebio.com/
