# Supplementary material for: The Active Components of Fuzheng Huayu Formula and Their Potential Mechanism of Action in Inhibiting the Hepatic Stellate Cells Viability – A Network Pharmacology and Transcriptomics Approach
Source: Front Pharmacol. 2018 May 24;9:525. doi: 10.3389/fphar.2018.00525 (PMC5976863; doi:10.3389/fphar.2018.00525)
Supplement: Supplementary file 1 [file Table_1.DOCX]

Supplementary Material

**The active components of Fuzheng Huayu formula and their potential mechanism of action in inhibiting the hepatic stellate cells viability - a network pharmacology and transcriptomics approach**

**Xinrui Xing^1†^, Si Chen^1,2^** **^†^, Ling Li^1†^, Yan Cao^1^, Langdong Chen^1^, Xiaobo Wang^2*^, Zhenyu Zhu^1*^**

^1^School of pharmacy, Second Military Medical University, Shanghai 200433, China

^2^Postdoctoral Research Workstation, 210th Hospital of the Chinese People’ s Liberation Army, Dalian 116021, China

^*^Correspondence:

Xiaobo Wang, wxbbenson0653@sina.com, Zhenyu Zhu, zzyzyfzhu@163.com

^†^These authors have contributed equally to this work.





**Figure S1**. The chemical structures of potential active components in FZHY formula.


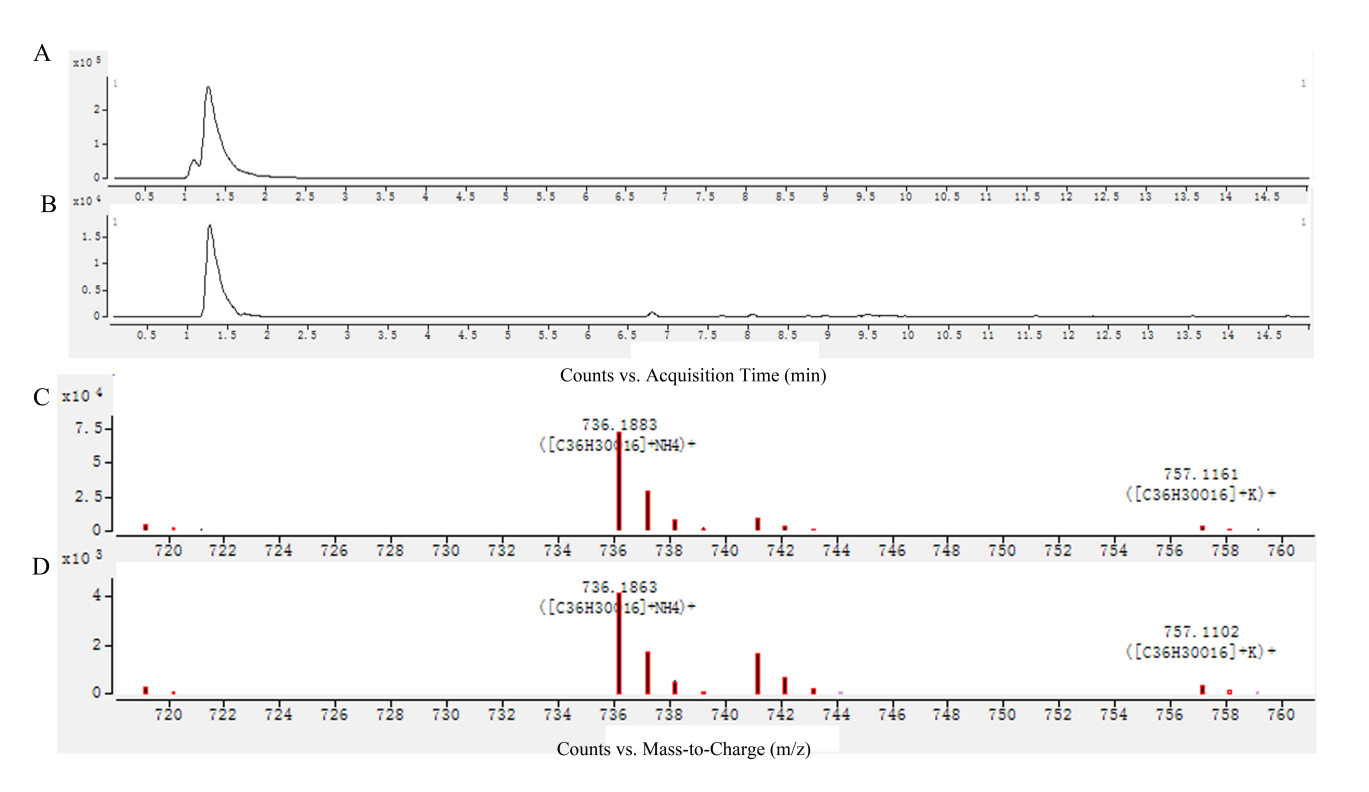


**Figure S2**. Identification of salvianolic acid B in FZHY formula. (A) Extracted ion chromatogram (EIC) of m/z 197.0448 in FZHY formula sample; (B ) Extracted ion chromatogram (EIC) of m/z 197.0449 of a commercial standard; (C) MS/MS spectrum of the ion in FZHY formula sample; (D) MS/MS spectrum of a commercial standard. The collision energy was 15 V.


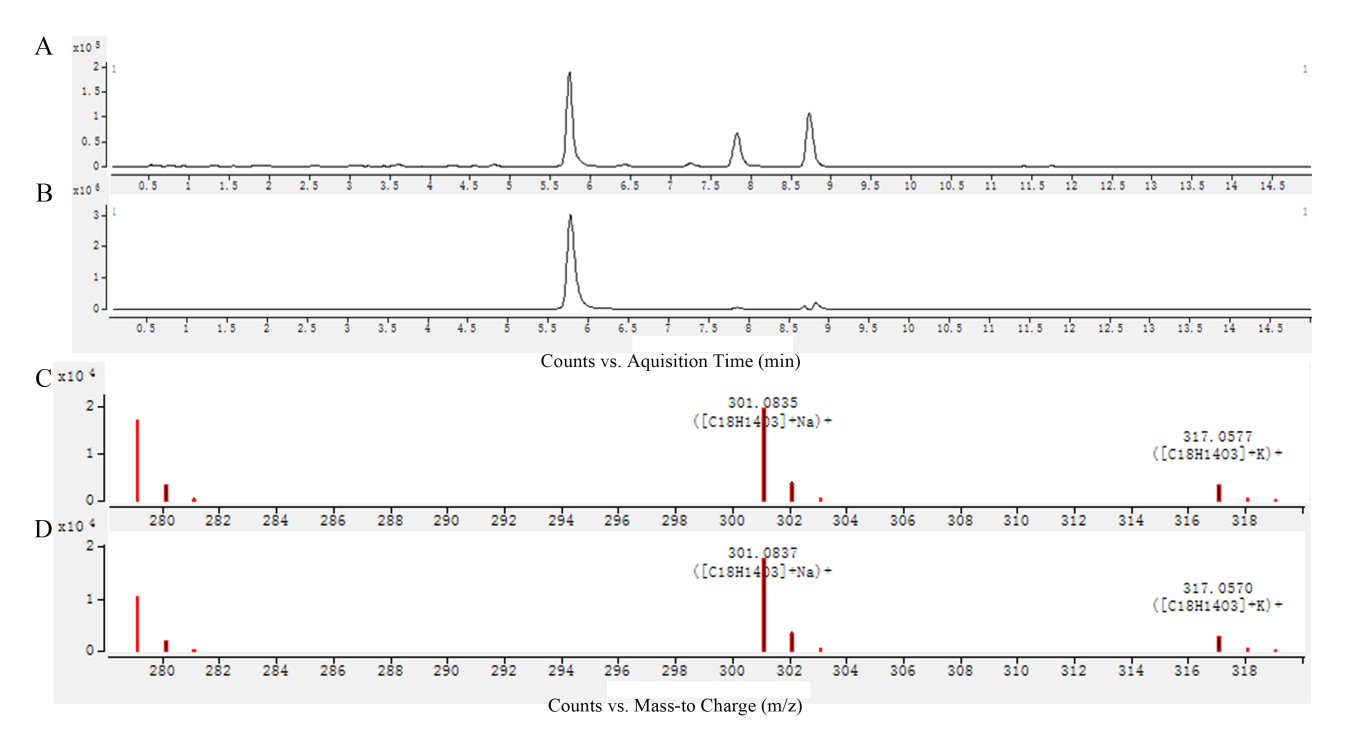


**Figure S3**. Identification of dihydrotanshinone I in FZHY formula. (A) Extracted ion chromatogram (EIC) of m/z 301.0835 in FZHY formula sample; (B ) Extracted ion chromatogram (EIC) of m/z 301.0837 of a commercial standard; (C) MS/MS spectrum of the ion in FZHY formula sample; (D) MS/MS spectrum of a commercial standard. The collision energy was 15 V.


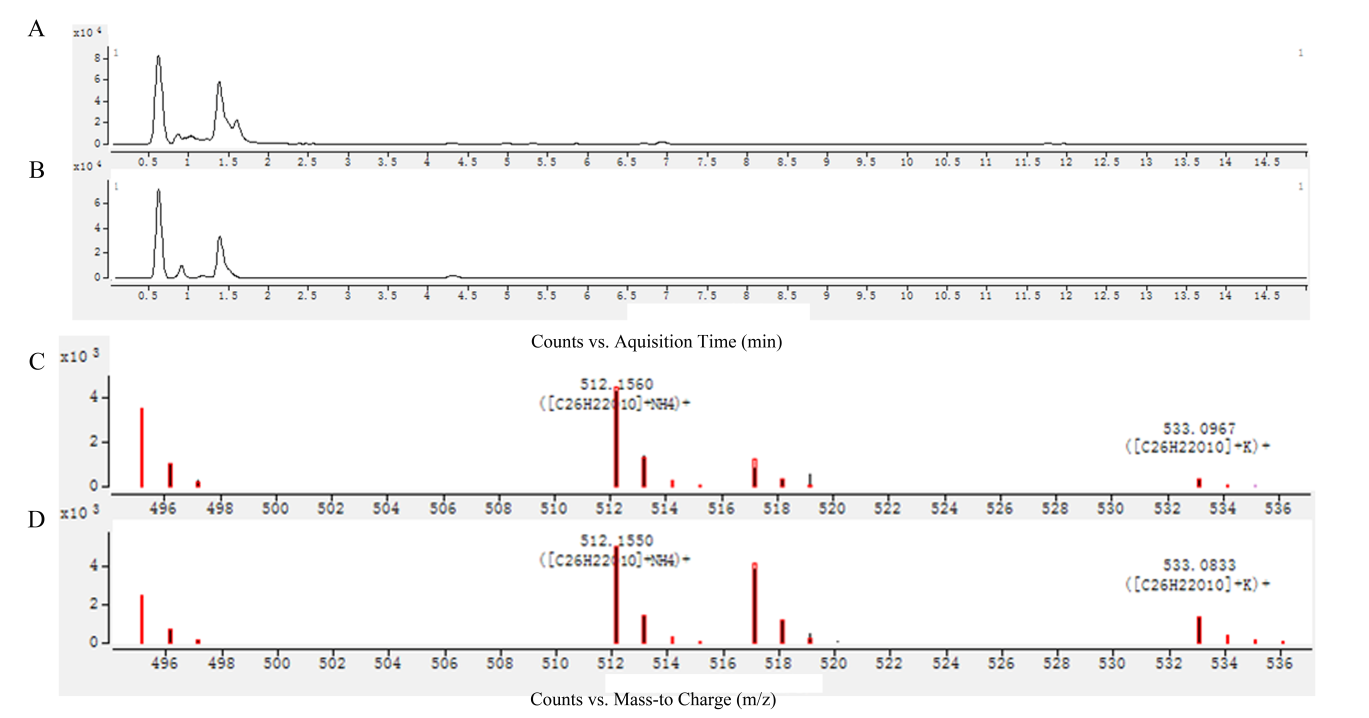


**Figure S4**. Identification of salvianolic acid A in FZHY formula. (A) Extracted ion chromatogram (EIC) of m/z 512.1560 in FZHY formula sample; (B ) Extracted ion chromatogram (EIC) of m/z 512.1550 of a commercial standard; (C) MS/MS spectrum of the ion in FZHY formula sample; (D) MS/MS spectrum of a commercial standard. The collision energy was 15 V.


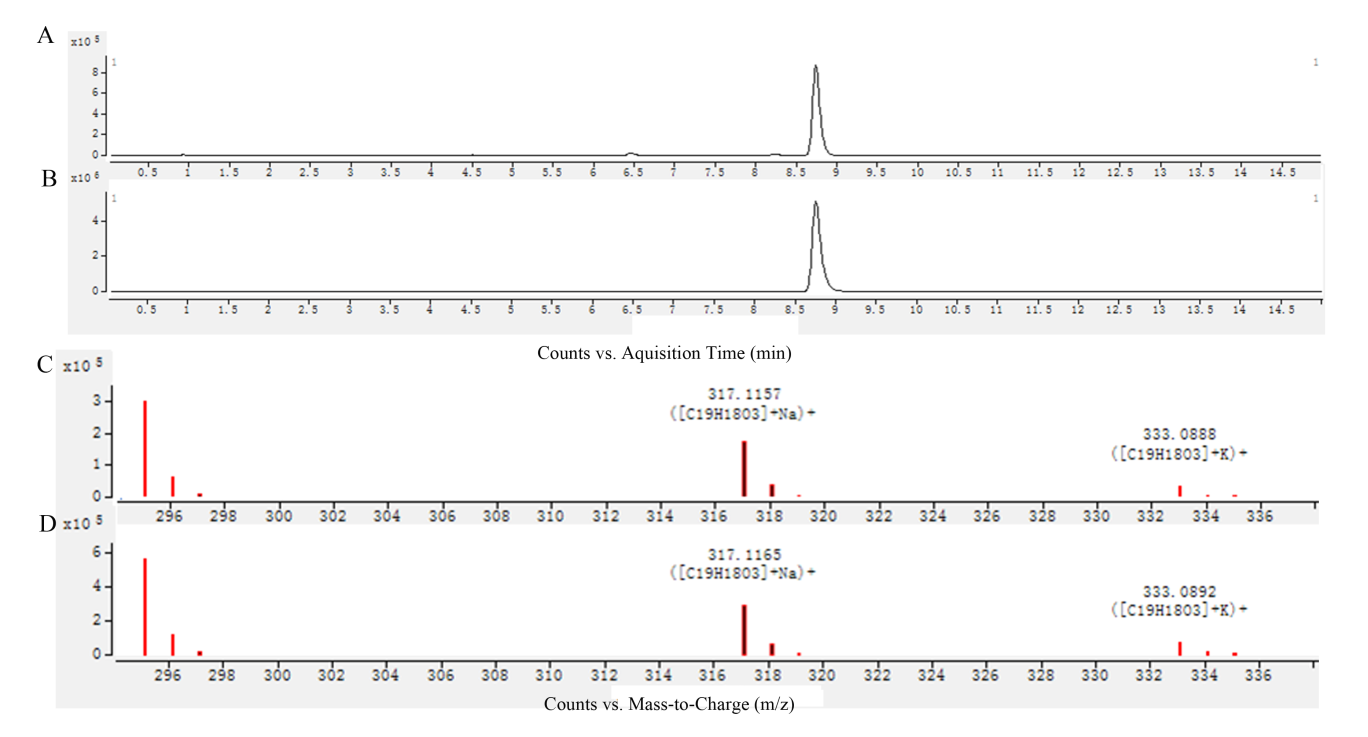


**Figure S5**. Identification of tanshinone-ⅡA in FZHY formula. (A) Extracted ion chromatogram (EIC) of m/z 317.1157 in FZHY formula sample; (B ) Extracted ion chromatogram (EIC) of m/z 317.1165 of a commercial standard; (C) MS/MS spectrum of the ion in FZHY formula sample; (D) MS/MS spectrum of a commercial standard. The collision energy was 15 V.


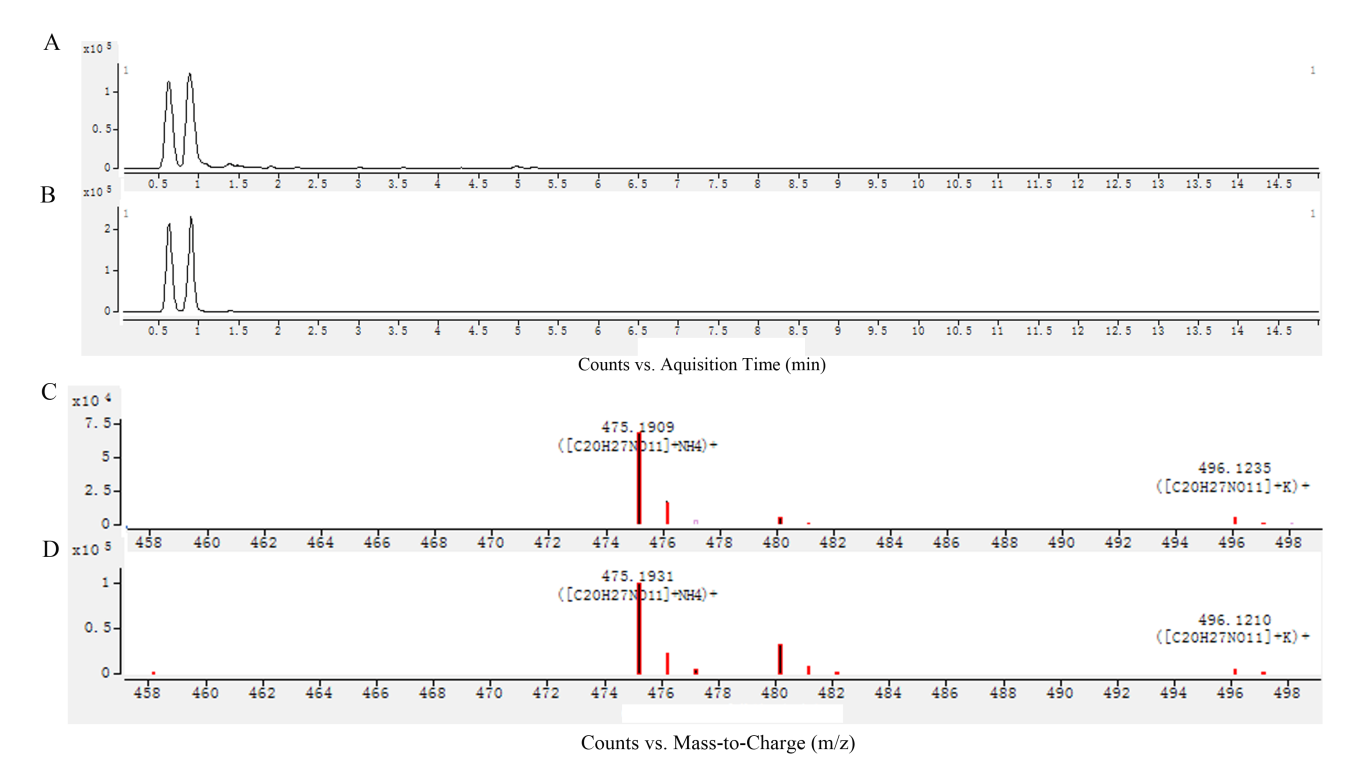


**Figure S6**. Identification of amygdalin in FZHY formula. (A) Extracted ion chromatogram (EIC) of m/z 475.1909 in FZHY formula sample; (B ) Extracted ion chromatogram (EIC) of m/z 475.1931 of a commercial standard; (C) MS/MS spectrum of the ion in FZHY formula sample; (D) MS/MS spectrum of a commercial standard. The collision energy was 15 V.


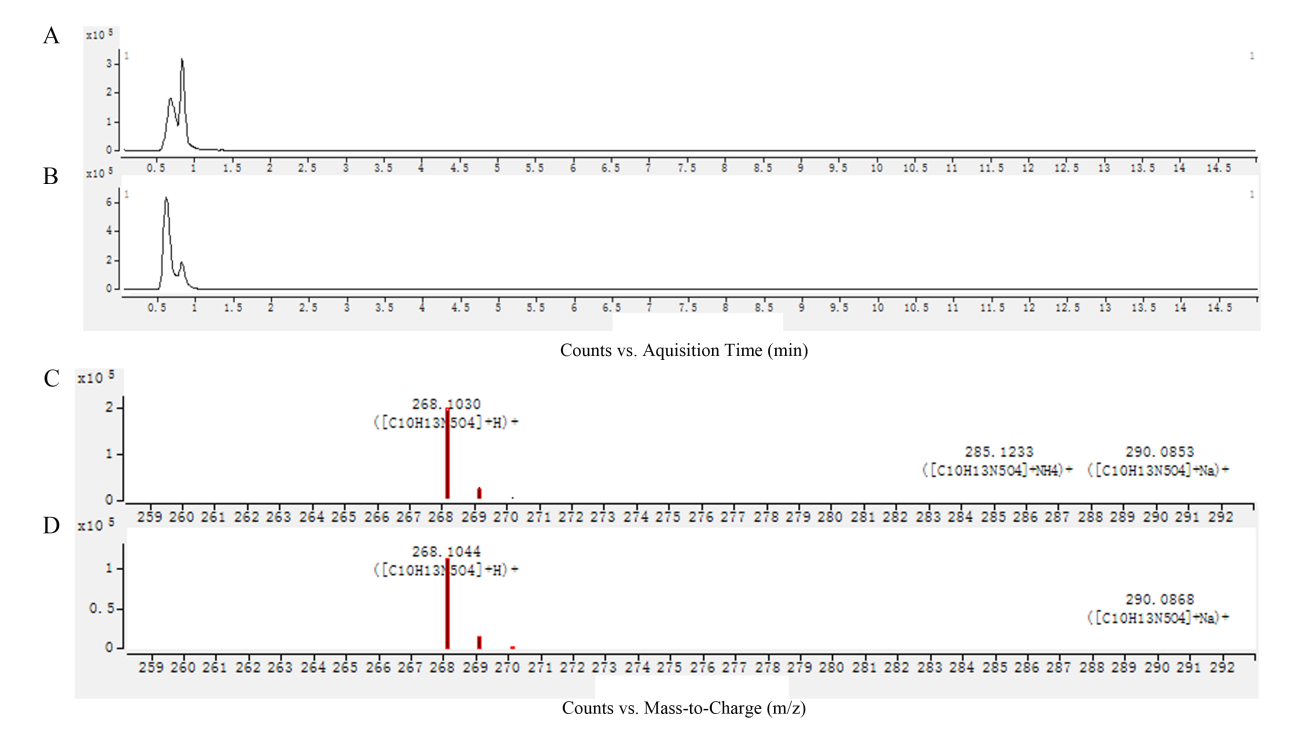


**Figure S7**. Identification of adenosine in FZHY formula. (A) Extracted ion chromatogram (EIC) of m/z 268.1030 in FZHY formula sample; (B ) Extracted ion chromatogram (EIC) of m/z 268.1044 of a commercial standard; (C) MS/MS spectrum of the ion in FZHY formula sample; (D) MS/MS spectrum of a commercial standard. The collision energy was 15 V.


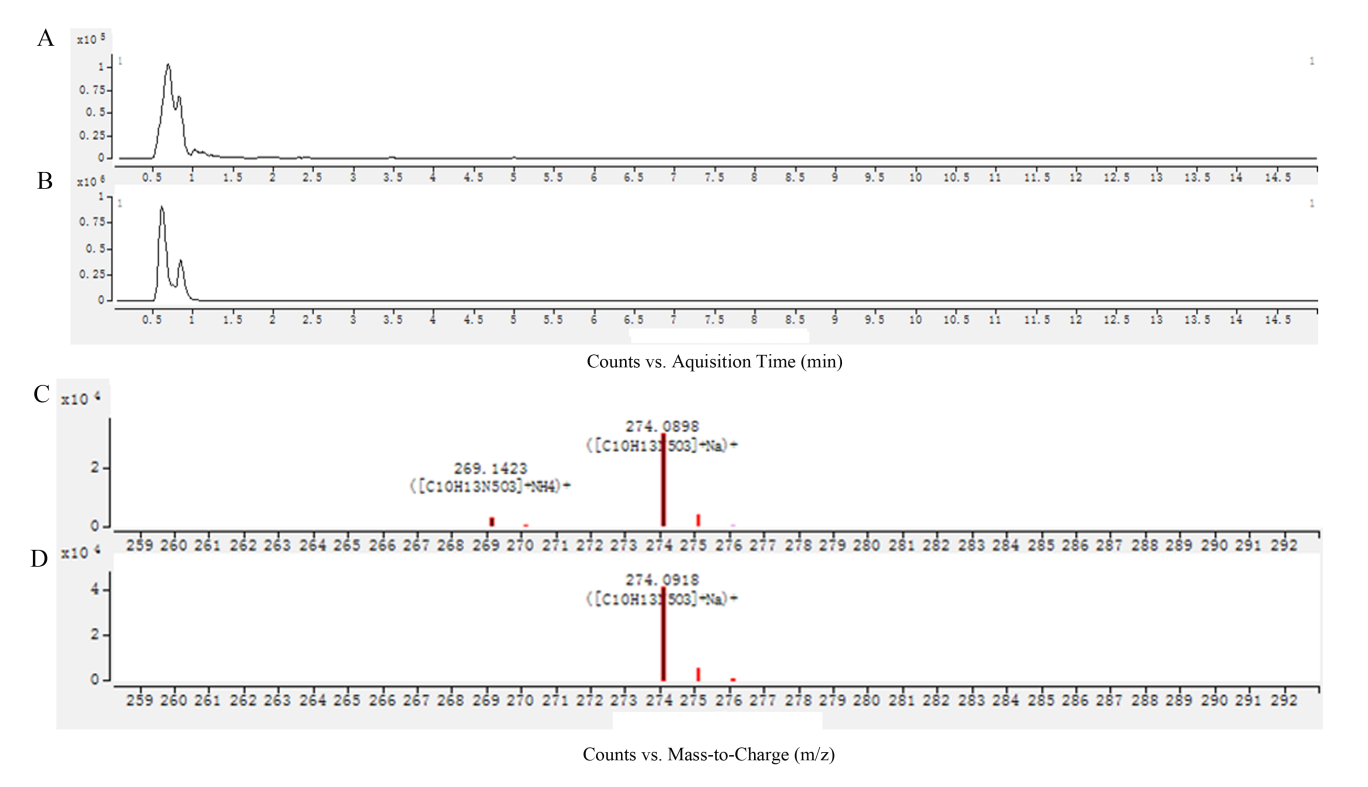


**Figure S8**. Identification of cordycepin in FZHY formula. (A) Extracted ion chromatogram (EIC) of m/z 274.0898 in FZHY formula sample; (B ) Extracted ion chromatogram (EIC) of m/z 274.0918 of a commercial standard; (C) MS/MS spectrum of the ion in FZHY formula sample; (D) MS/MS spectrum of a commercial standard. The collision energy was 15 V.


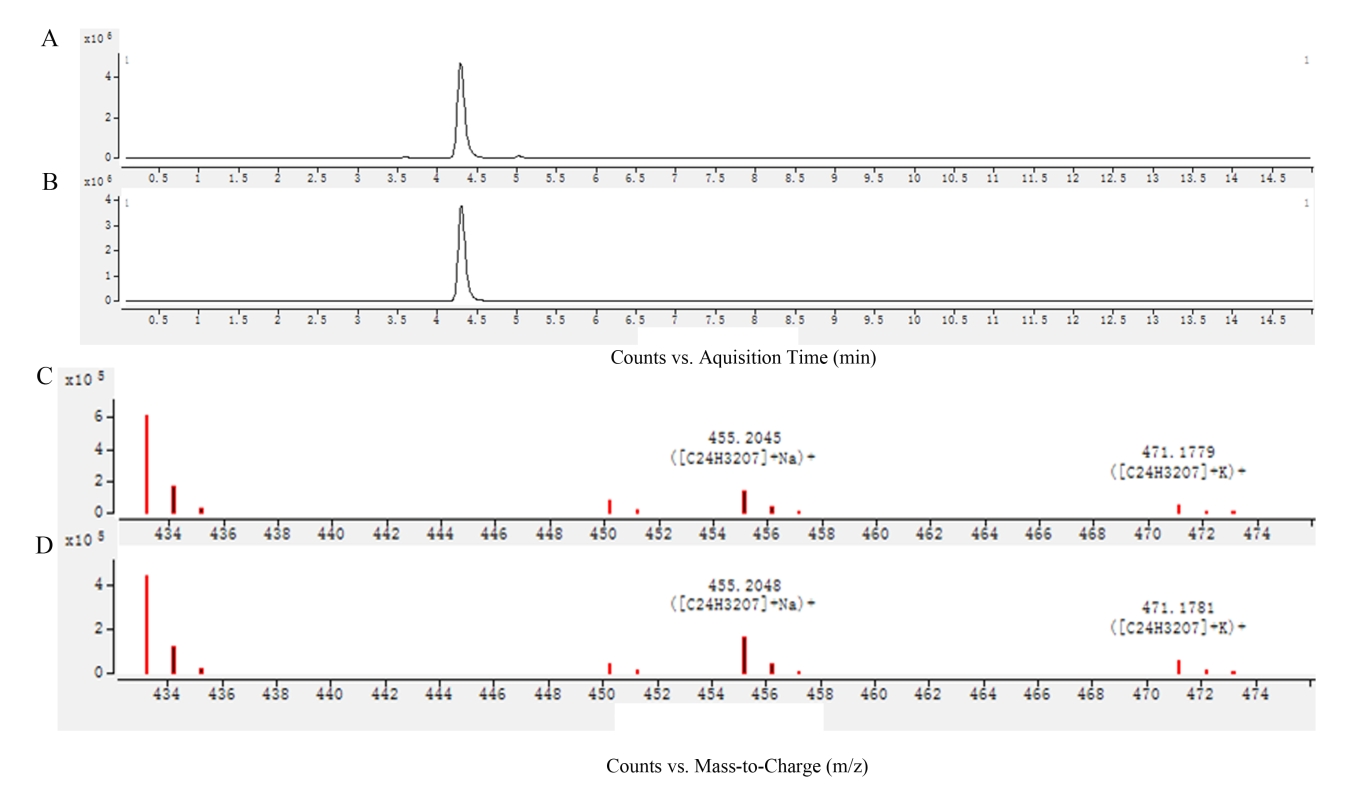


**Figure S9**. Identification of schizandrin in FZHY formula. (A) Extracted ion chromatogram (EIC) of m/z 455.2045 in FZHY formula sample; (B ) Extracted ion chromatogram (EIC) of m/z 455.2048of a commercial standard; (C) MS/MS spectrum of the ion in FZHY formula sample; (D) MS/MS spectrum of a commercial standard. The collision energy was 15 V.


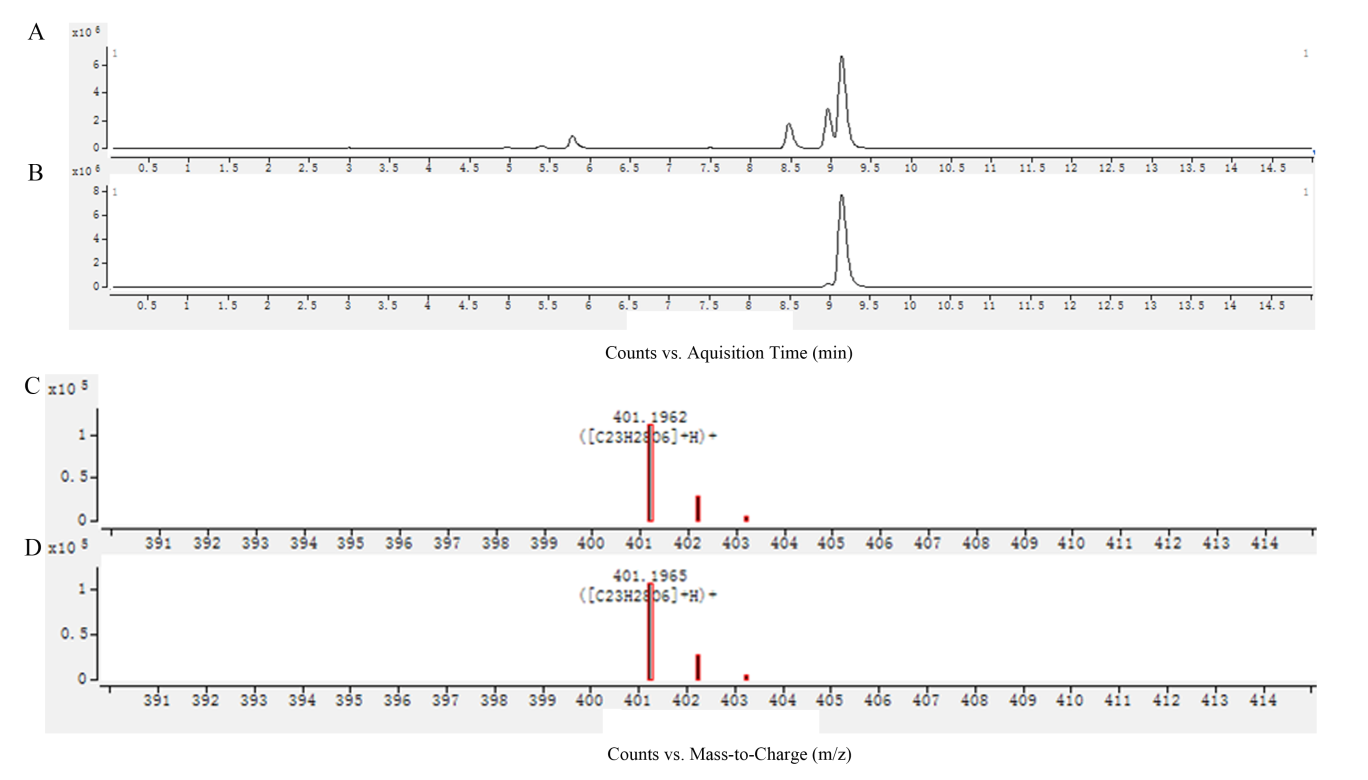


**Figure S10**. Identification of schisandrin B in FZHY formula. (A) Extracted ion chromatogram (EIC) of m/z 401.1962 in FZHY formula sample; (B ) Extracted ion chromatogram (EIC) of m/z 401.1965 of a commercial standard; (C) MS/MS spectrum of the ion in FZHY formula sample; (D) MS/MS spectrum of a commercial standard. The collision energy was 15 V.


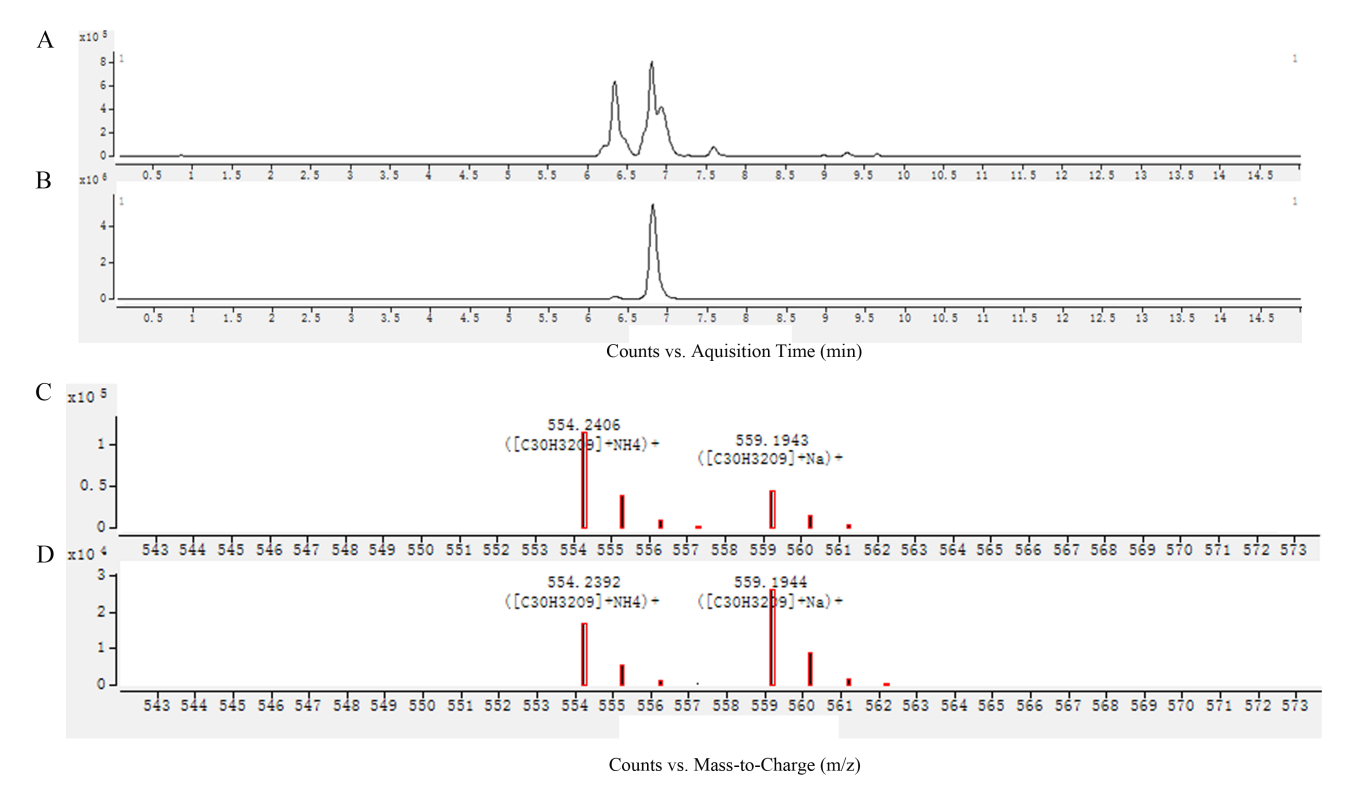


**Figure S11**. Identification of schisantherin A in FZHY formula. (A) Extracted ion chromatogram (EIC) of m/z 554.2406 in FZHY formula sample; (B ) Extracted ion chromatogram (EIC) of m/z 554.2392 of a commercial standard; (C) MS/MS spectrum of the ion in FZHY formula sample; (D) MS/MS spectrum of a commercial standard. The collision energy was 15 V.


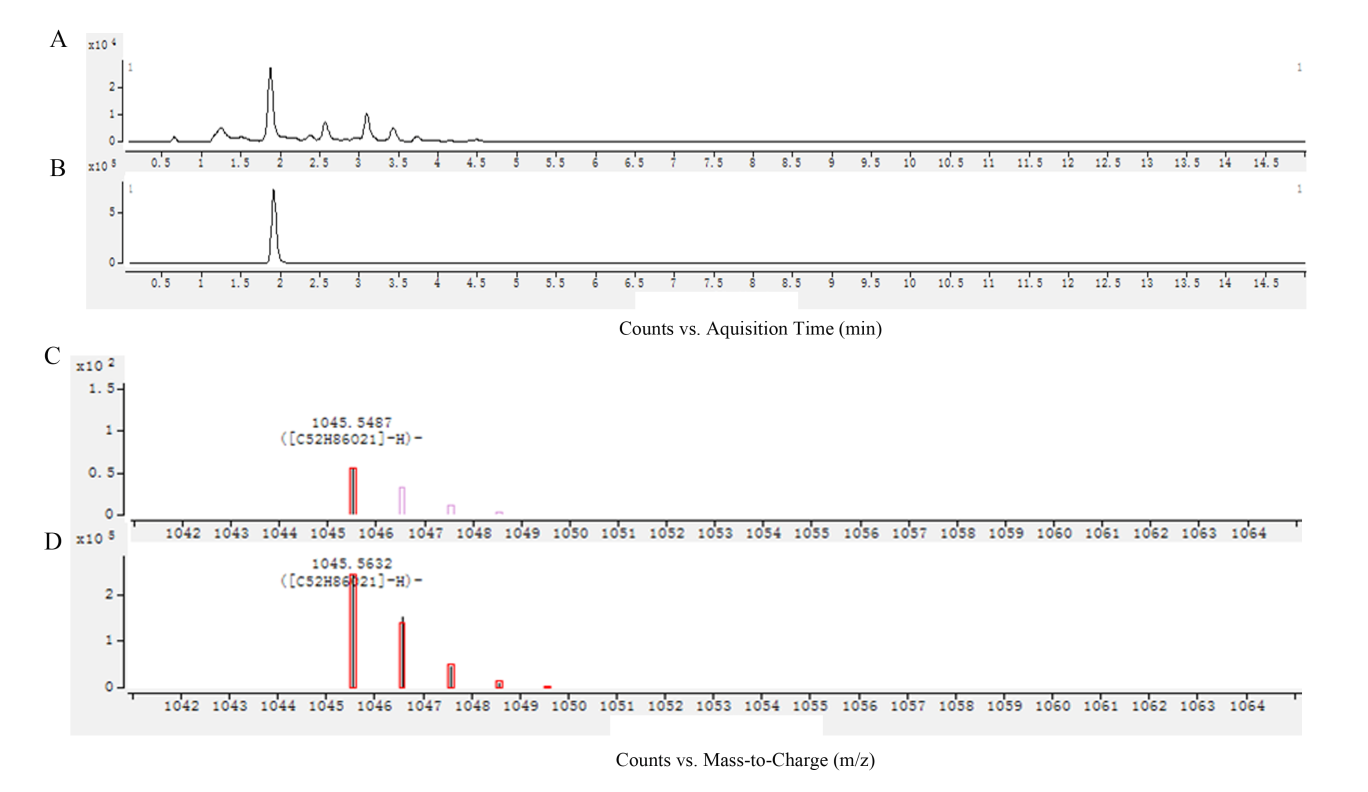


**Figure S12**. Identification of gypenoside XLIX in FZHY formula. (A) Extracted ion chromatogram (EIC) of m/z 1045.5487 in FZHY formula sample; (B ) Extracted ion chromatogram (EIC) of m/z 1045.5632 of a commercial standard; (C) MS/MS spectrum of the ion in FZHY formula sample; (D) MS/MS spectrum of a commercial standard. The collision energy was 15 V.


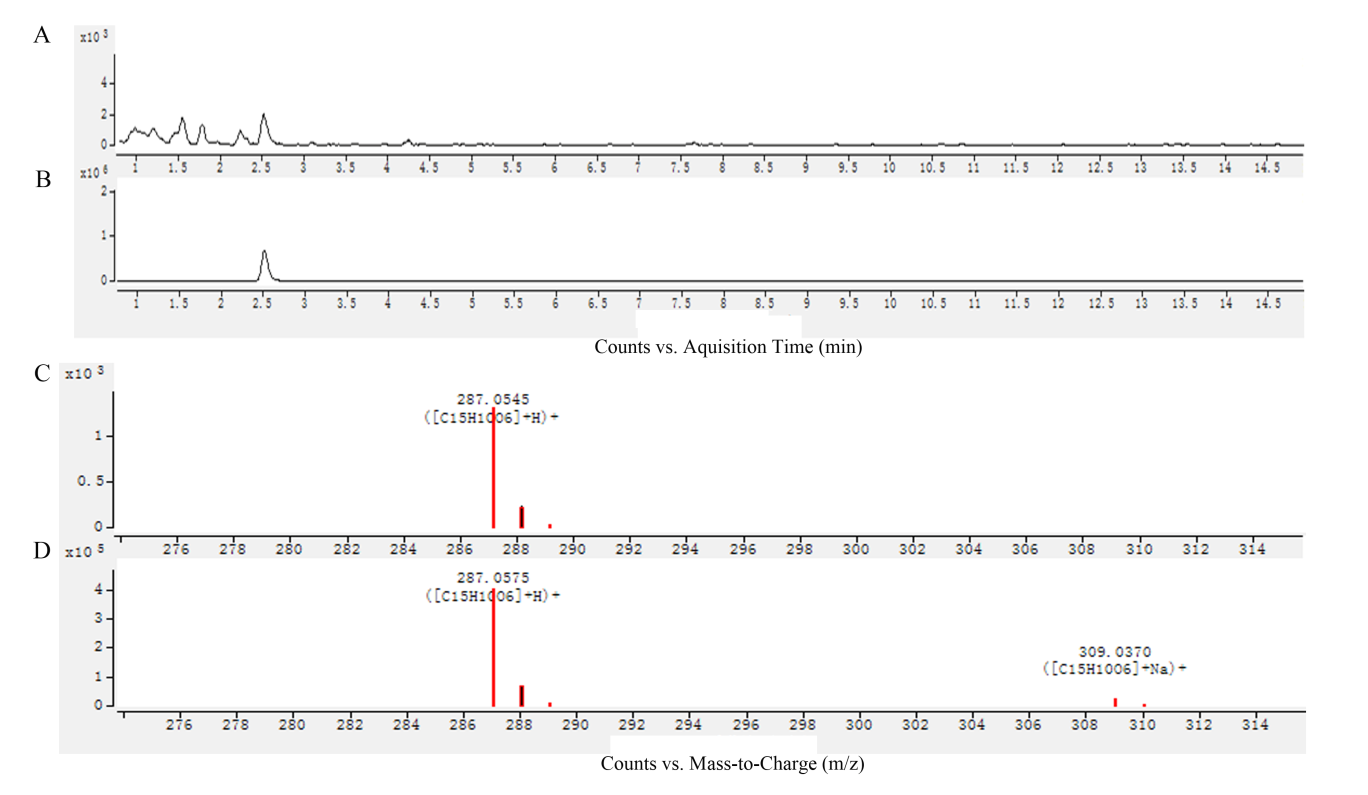


**Figure S13**. Identification of kaempferol in FZHY formula. (A) Extracted ion chromatogram (EIC) of m/z 287.0545 in FZHY formula sample; (B ) Extracted ion chromatogram (EIC) of m/z 287.0575of a commercial standard; (C) MS/MS spectrum of the ion in FZHY formula sample; (D) MS/MS spectrum of a commercial standard. The collision energy was 15 V.


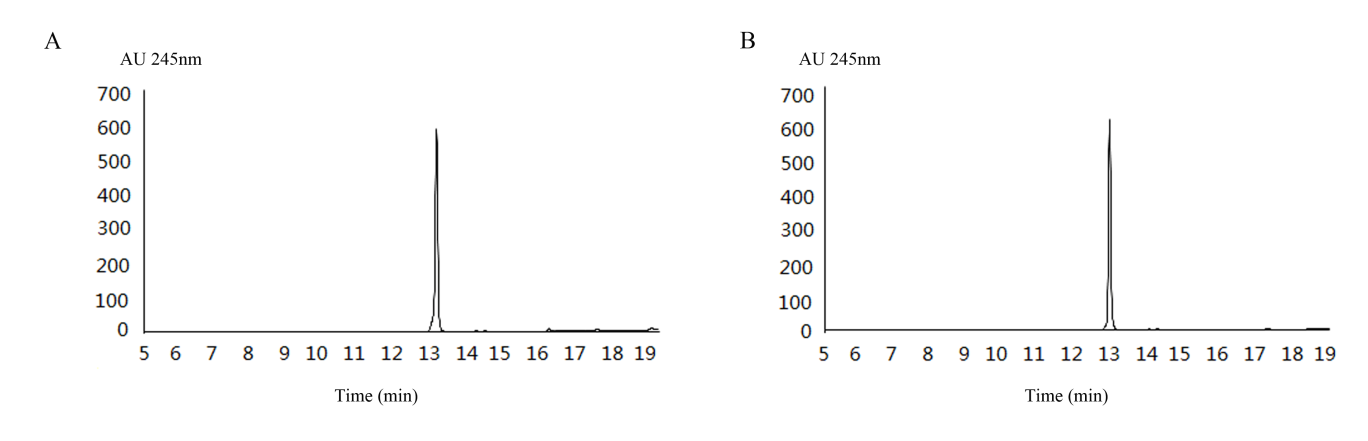


**Figure S14**. Analytical injections were monitored at 254 nm. (A) HPLC chromatograms of salvianolic acid B. (B) HPLC chromatograms of salvianolic acid B that was incubated in DMSO for 24 hours.


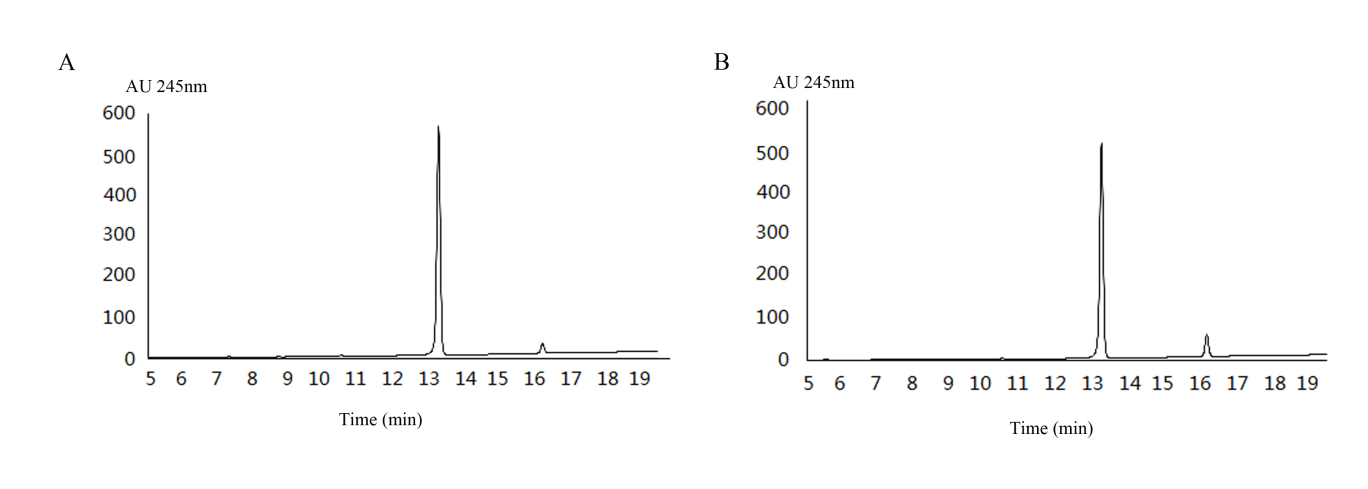


**Figure S15**. Analytical injections were monitored at 254 nm. (A) HPLC chromatograms of dihydrotanshinone I. (B) HPLC chromatograms of dihydrotanshinone I that was incubated in DMSO for 24 hours.


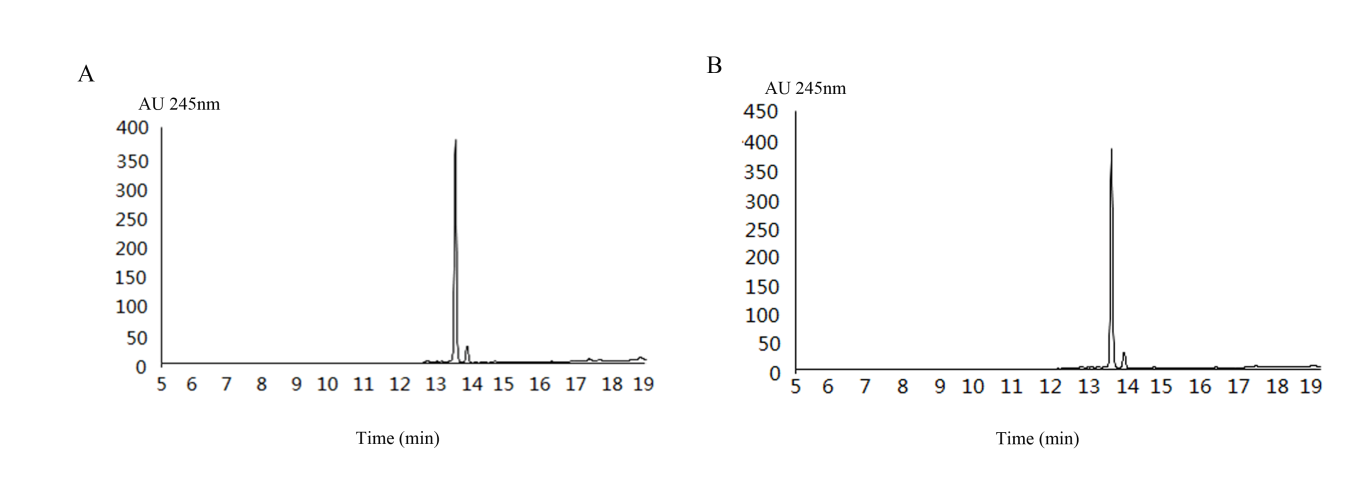


**Figure S16**. Analytical injections were monitored at 254 nm. (A) HPLC chromatograms of salvianolic acid A. (B) HPLC chromatograms of salvianolic acid A that was incubated in DMSO for 24 hours.


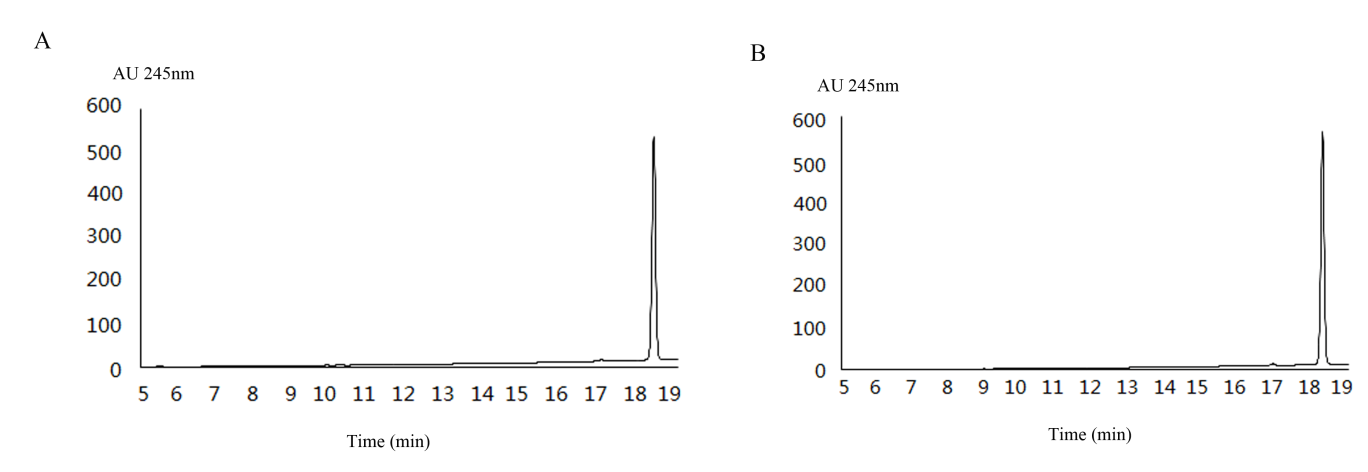


**Figure S17**. Analytical injections were monitored at 254 nm. (A) HPLC chromatograms of tanshinone-ⅡA. (B) HPLC chromatograms of tanshinone-ⅡA that was incubated in DMSO for 24 hours.


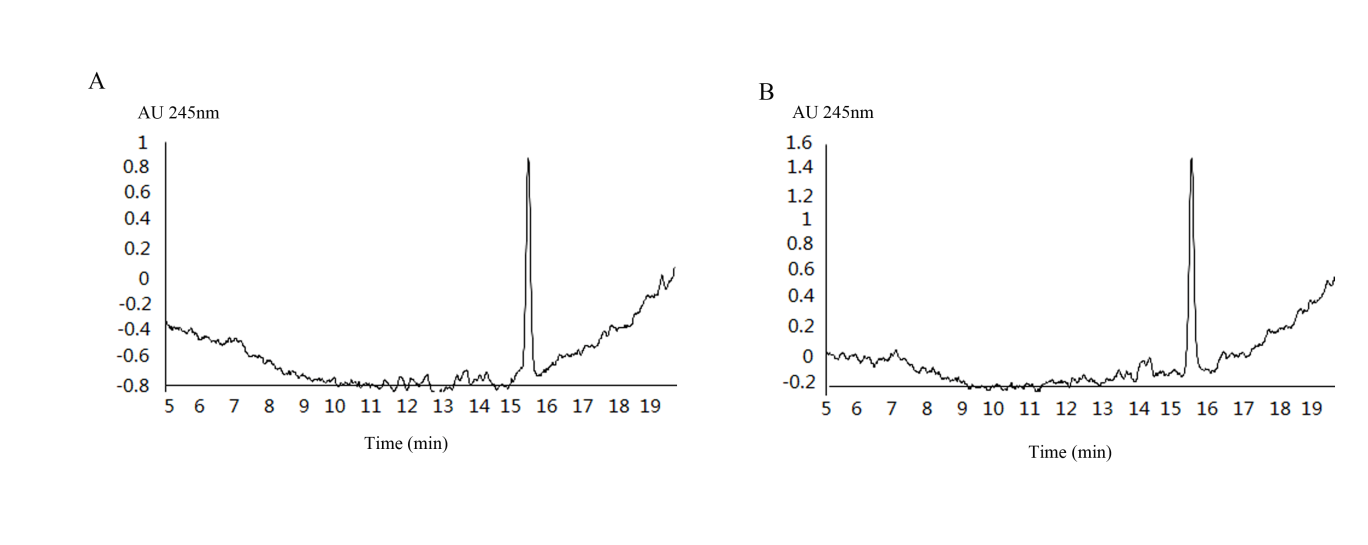


**Figure S18**. Analytical injections were monitored at 254 nm. (A) HPLC chromatograms of cordycepin. (B) HPLC chromatograms of cordycepin that was incubated in DMSO for 24 hours.


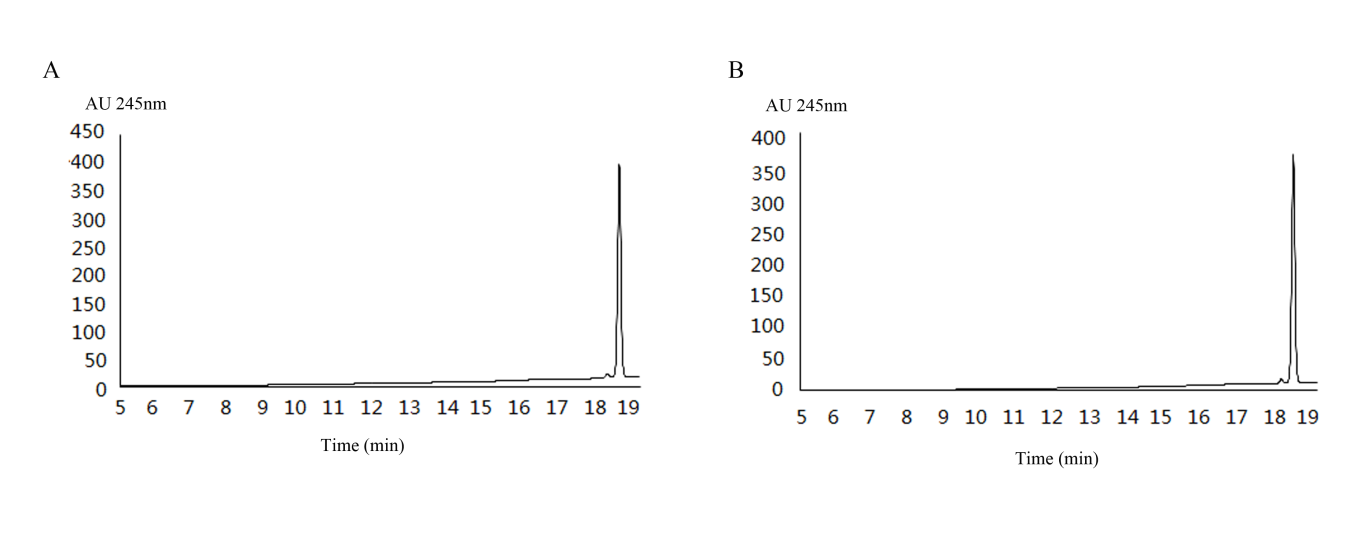


**Figure S19**. Analytical injections were monitored at 254 nm. (A) HPLC chromatograms of schisandrin B. (B) HPLC chromatograms of schisandrin B that was incubated in DMSO for 24 hours.


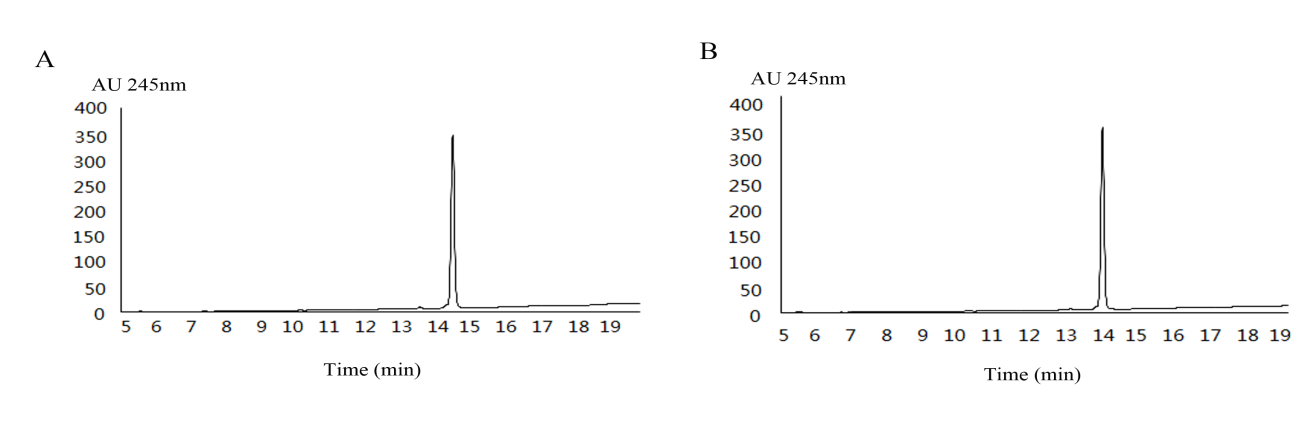


**Figure S20**. Analytical injections were monitored at 254 nm. (A) HPLC chromatograms of schisantherin A. (B) HPLC chromatograms of schisantherin A that was incubated in DMSO for 24 hours.


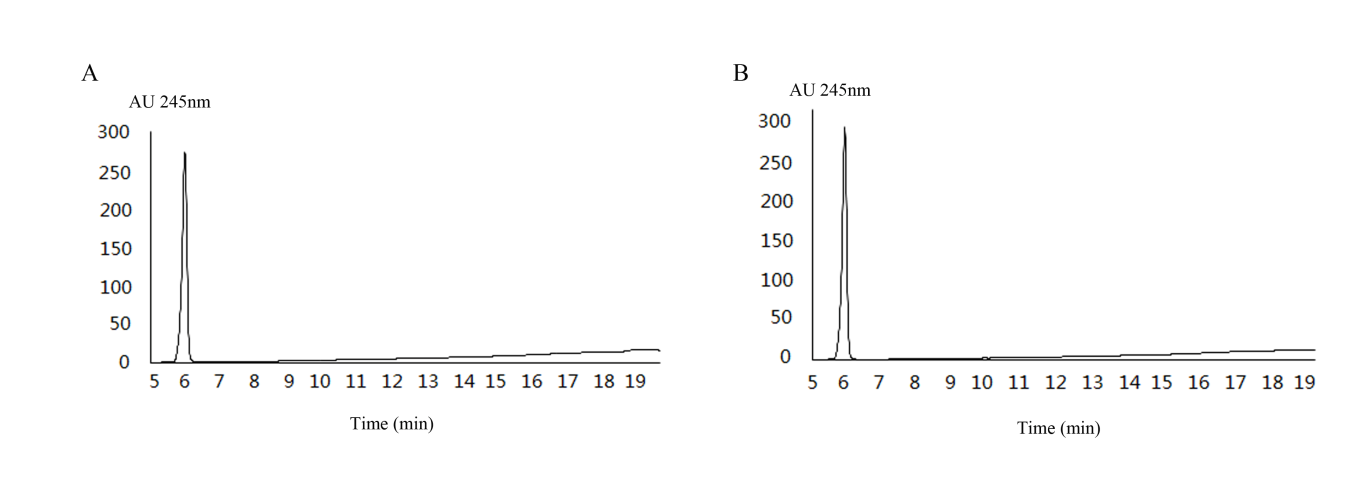


**Figure S21**. Analytical injections were monitored at 254 nm. (A) HPLC chromatograms of kaempferol. (B) HPLC chromatograms of kaempferol that was incubated in DMSO for 24 hours.

**Table S1.** Targets were screened by similarity and molecular docking.

| No | Target | Origin | No | Target | Origin | No | Target | Origin |
| --- | --- | --- | --- | --- | --- | --- | --- | --- |
| 1 | MEN1 | similarity | 28 | PERM | similarity | 55 | NISCH | docking |
| 2 | PGH2 | similarity | 29 | PPARD | similarity | 56 | RAGE | docking |
| 3 | DPP4 | similarity | 30 | PPARG | similarity | 57 | RHOA | docking |
| 4 | MMP2 | similarity | 31 | SGK1 | similarity | 58 | PAK1 | docking |
| 5 | NR1I2 | similarity | 32 | STK25 | similarity | 59 | RORG | docking |
| 6 | STAT3 | similarity | 33 | TPO | similarity | 60 | 2ABA | docking |
| 7 | AKT1 | similarity | 34 | UROK | similarity | 61 | CCR9 | docking |
| 8 | CAH3 | similarity | 35 | VGFR2 | similarity | 62 | CYGB | docking |
| 9 | CAH9 | similarity | 36 | GCR | similarity | 63 | TGM2 | docking |
| 10 | CHLE | similarity | 37 | PPARA | similarity | 64 | TYB4 | docking |
| 11 | CP1A2 | similarity | 38 | TF | similarity | 65 | VDR | docking |
| 12 | CP2D6 | similarity | 39 | EZH2 | similarity | 66 | SIR1 | docking |
| 13 | EGFR | similarity | 40 | HDAC4 | similarity | 67 | SMAD2 | docking |
| 14 | ESR1 | similarity | 41 | HDAC7 | similarity | 68 | SMAD4 | docking |
| 15 | FGFR2 | similarity | 42 | HDAC1 | similarity | 69 | STAT1 | docking |
| 16 | HS90B | similarity | 43 | CATL1 | similarity | 70 | ASM | docking |
| 17 | IGF1R | similarity | 44 | PDCD4 | similarity | 71 | BRD4 | docking |
| 18 | JUN | similarity | 45 | PGFRB | similarity | 72 | CCL2 | docking |
| 19 | KKCC2 | similarity | 46 | IL17 | similarity | 73 | CCL3 | docking |
| 20 | KS6B1 | similarity | 47 | PTN1 | docking | 74 | HIF1A | docking |
| 21 | LOX15 | similarity | 48 | M3K7 | docking | 75 | HB-EGF | docking |
| 22 | MK08 | similarity | 49 | PTEN | docking | 76 | GDF2 | docking |
| 23 | MMP12 | similarity | 50 | 5NTD | docking | 77 | MECP2 | docking |
| 24 | MTOR | similarity | 51 | ABL1 | docking | 78 | S10A4 | docking |
| 25 | NUAK1 | similarity | 52 | ACE2 | docking | 79 | VHL | docking |
| 26 | P53 | similarity | 53 | NEDD8 | docking |  |  |  |
| 27 | PA21B | similarity | 54 | NF2L2 | docking |  |  |  |

**Table S2.** The known targets of liver fibrosis.

| No | Targets | No | Targets |
| --- | --- | --- | --- |
| 1 | C-C motif chemokine receptor (CCR) 9 | 37 | Smad2 |
| 2 | Acidic sphingomyelinase (ASM) | 38 | Vitamin D receptor (VDR) ligands |
| 3 | Tyrosine kinase Abl1 | 39 | Protein tyrosine phosphatase 1B (PTP1B) |
| 4 | Hepatic IL-17A | 40 | WNT-inducible signaling pathway protein-1 (WISP-1) |
| 5 | CC chemokine L3（CCL3） | 41 | TRPM7, a non-selective cation channel of the TRP channel superfamily |
| 6 | RhoA | 42 | Transmembrane 4 L6 family member 5 (TM4SF5) |
| 7 | Phosphatase and tensin homolog deleted on chromosome 10 (PTEN) | 43 | Hsp47 (heat shock protein 47) |
| 8 | Methyl-CpG-binding protein 2 (MeCP2) | 44 | Transient receptor potential melastatin 7 (TRPM7) |
| 9 | Signal transducer and activator of transcription 3 (STAT3) | 45 | Sphingosine 1-phosphate (S1P) |
| 10 | Bone morphogenetic protein 9 (BMP9) | 46 | Retinol dehydrogenase 13 (RDH13) |
| 11 | Tissue transglutaminase (TG2) | 47 | Periostin |
| 12 | Thymosin beta-4 (Tbeta4) | 48 | Protease-activated receptor-2 (PAR2) |
| 13 | P21-activated kinase (PAK) | 49 | Purinergic P2X7R |
| 14 | RAR-related orphan receptor gamma (ROR-gamma) | 50 | NS5ATP13 (VIRAL HEPATITIS TYPE C NS5A-transactivated protein 13) |
| 15 | The c-Jun N-terminal kinase-1 (Jnk1) | 51 | Hyaluronan synthase 3 |
| 16 | SIRT1 (silent information regulator 1), | 52 | Homeodomain-interacting protein kinase 2 (HIPK2) |
| 17 | Hypoxia-inducible factor (HIF) | 53 | connexin32 |
| 18 | Ecto-5'-nucleotidase/CD73 enzyme | 54 | Connective tissue growth factor (CTGF) |
| 19 | TGF-beta activated kinase 1 (TAK1) | 55 | CD248 (endosialin) |
| 20 | Monocyte chemoattractant protein-1 （MCP-1） | 56 | C1q/tumor necrosis factor-related protein 3 (CTRP3） |
| 21 | Von Hippel-Lindau protein (VHL), | 57 | Apelin-APJ axis /G-protein-coupled receptor APJ |
| 22 | Activator of transcription 1 (STAT1) | 58 | 5-HT7 receptor agonist 、 LP-44 |
| 23 | 70-kDa ribosomal S6 kinase (p70S6K) | 59 | Hydrogen peroxide-inducible clone-5 (Hic-5)/ transforming growth factor beta-1-induced transcript 1 protein (Tgfb1i1) |
| 24 | Imidazoline I1 receptor (I1R) | 60 | Y-box binding protein (YB-1) |
| 25 | Receptor for advanced glycation end products (RAGE)-ligand axis | 61 | Nucleolin (NCL) |
| 26 | Platelet-derived growth factor receptor-beta (PDGFR-beta | 62 | High-mobility group box 1 (HMGB1) |
| 27 | Peroxisome proliferator-activated receptor-gamma (PPARgamma) | 63 | Early growth response-1(Egr-1) |
| 28 | S100A4 | 64 | Stromal cell-derived factor-1 (SDF-1) |
| 29 | Nedd8(Neddylation) | 65 | Thymosin beta 4 (Tbeta4) |
| 30 | Protein phosphatase 2A | 66 | RACK1, the receptor for activated C-kinase 1, |
| 31 | Bromodomain-containing protein 4 (BRD4) | 67 | HAb18G/CD147, a tumor-related glycoprotein, |
| 32 | Nuclear erythroid 2-related factor 2 (Nrf2) | 68 | Toll-like receptor 3 (TLR3) |
| 33 | Cytoglobin | 69 | Secreted protein, acidic and rich in cysteine (SPARC) |
| 34 | SMAD4. | 70 | Orphan nuclear receptor(NR4A2 ) |
| 35 | Heparin-binding epidermal growth factor-like growth factor (HB-EGF) | 71 | Smad3 |
| 36 | Angiotensin converting enzyme 2 (ACE2) |  |  |

**Table S3.** Main biological process significantly relating to targets.

| #pathway ID | Biological process description | targets |
| --- | --- | --- |
| GO.0071495 | cellular response to endogenous stimulus | ABL1,AKT1,ESR1,FGFR2,HBEGF,HDAC1,  HDAC4,IGF1R,IL17A,JUN,MMP2,MTOR,  NEDD8,NR1I2,NR3C1,PPARA,PPARD,  PPARG,PTEN,RHOA,RORC,SMAD2,SMAD4,  STAT1,STAT3,TP53,VDR |
| GO.0010557 | positive regulation of macromolecule biosynthetic process | AKT1,BRD4,CCL2,EGFR,ESR1,FGFR2,HDAC1,  HDAC4,HIF1A,IGF1R,IL17A,JUN,MECP2,  MEN1,MTOR,NR1I2,NR3C1,PPARA,PPARD,  PPARG,PTEN,RORC,SMAD2,SMAD4,STAT1,  STAT3,TF,TP53,VDR,VHL |
| GO.0009719 | response to endogenous stimulus | ABL1,AKT1,ESR1,EZH2,FGFR2,HBEGF,HDAC1,  HDAC4,IGF1R,IL17A,JUN,MMP2,MTOR,  NEDD8,NR1I2,NR3C1,PPARA,PPARD,PPARG,  RHOA,RORC,SMAD2,SMAD4,SMPD1,STAT1,  STAT3,TP53,VDR |
| GO.0010941 | regulation of cell death | AKT1,CCL2,CCL3,EGFR,ESR1,FGFR2,HDAC1,  HDAC4,IL17A,MECP2,NR3C1,PAK1,PDCD4,  PPARA,PPARG,PTEN,RORC,SGK1,SMAD4,  SMPD1,STAT1,STAT3,STK25,TGM2,TIMP1,  TP53,VDR,VHL |
| GO.0045935 | positive regulation of nucleobase-containing compound metabolic process | AKT1,BRD4,EGFR,ESR1,FGFR2,HDAC1,HDAC4,  HIF1A,IGF1R,IL17A,JUN,MECP2,MEN1,MTOR,  NR1I2,NR3C1,PPARA,PPARD,PPARG,PTEN,  RORC,SMAD2,SMAD4,STAT1,STAT3,TF,TP53,  VDR,VHL |
| GO.0006367 | transcription initiation from RNA polymerase II promoter | AKT1,ESR1,HDAC1,MEN1,MTOR,NR1I2,  NR3C1,PPARA,PPARD,PPARG,PTEN,RORC,  SMAD2,SMAD4,TP53,VDR |
| GO.0010604 | positive regulation of macromolecule metabolic process | AKT1,BRD4,CCL2,CCL3,EGFR,ESR1,EZH2,  FGFR2,HDAC1,HDAC4,HIF1A,IGF1R,IL17A,  JUN,MECP2,MEN1,NR1I2,NR3C1,PAK1,  PPARA,PPARD,PPARG,PTEN,RORC,SMAD2,  SMAD4,SMPD1,STAT1,STAT3,STK25,TF,  TP53,VDR,VHL |
| GO.0032870 | cellular response to hormone stimulus | AKT1,CCL2,EGFR,ESR1,FGFR2,HDAC4,IGF1R,  IL17A,JUN,MEN1,MTOR,NR1I2,NR3C1,PPARA,  PPARD,PPARG,RORC,STAT1,STAT3,VDR |
| GO.0031328 | positive regulation of cellular biosynthetic process | AKT1,BRD4,EGFR,ESR1,FGFR2,HDAC1,HDAC4,  HIF1A,IGF1R,IL17A,JUN,MECP2,MEN1,MTOR,  NR1I2,NR3C1,PPARA,PPARD,PPARG,PTEN,  RORC,SMAD2,SMAD4,STAT1,STAT3,TF,TP53,  VDR,VHL |
| GO.0045893 | positive regulation of transcription, DNA-templated | AKT1,BRD4,EGFR,ESR1,FGFR2,HDAC1,HDAC4,  IL17A,JUN,MECP2,MEN1,MTOR,NR1I2,NR3C1,  PPARA,PPARD,PPARG,PTEN,RORC,SMAD2,  SMAD4,STAT1,STAT3,TF,TP53,VDR,VHL |

**Table S4.** Main pathway significantly relating to targets.

| #pathway ID | pathway description | targets | Degree |
| --- | --- | --- | --- |
| 4066 | HIF-1 signaling pathway | AKT1,EGFR,HIF1A,IGF1R,MTOR,  STAT3,TF,TIMP1,VHL | 8 |
| 4068 | FoxO signaling pathway | AKT1,EGFR,IGF1R,PTEN,SGK1,  SMAD2,SMAD4,STAT3 | 8 |
| 4012 | ErbB signaling pathway | ABL1,AKT1,EGFR,HBEGF,JUN,  MTOR,PAK1 | 7 |
| 4062 | Chemokine signaling pathway | AKT1,CCL2,CCL3,CCR9,PAK1,  RHOA,STAT1,STAT3 | 8 |
| 4919 | Thyroid hormone signaling pathway | AKT1,ESR1,HDAC1,HIF1A,MTOR,  STAT1,TP53 | 7 |
| 4915 | Estrogen signaling pathway | AKT1,EGFR,ESR1,HBEGF,JUN,  MMP2 | 6 |
| 4014 | Ras signaling pathway | ABL1,AKT1,EGFR,FGFR2,IGF1R,  PAK1,RHOA | 7 |
| 4151 | PI3K-Akt signaling pathway | AKT1,EGFR,FGFR2,IGF1R,MTOR,  PTEN,SGK1,TP53 | 8 |
| 4152 | AMPK signaling pathway | AKT1,CPT1C,IGF1R,MTOR,PPARG | 5 |
| 4150 | mTOR signaling pathway | AKT1,HIF1A,MTOR,PTEN | 4 |
| 3320 | PPAR signaling pathway | CPT1C,PPARA,PPARD,PPARG | 4 |
| 4010 | MAPK signaling pathway | AKT1,EGFR,FGFR2,JUN,PAK1,  TP53 | 6 |
| 4350 | TGF-beta signaling pathway | RHOA,SMAD2,SMAD4 | 3 |
| 4620 | Toll-like receptor signaling pathway | AKT1,CCL3,JUN,STAT1 | 4 |
| 4310 | Wnt signaling pathway | JUN,PPARD,RHOA,SMAD4,TP53 | 5 |

**Table S5.** Main cell components significantly relating to targets.

| #pathway ID | pathway description | targets |
| --- | --- | --- |
| GO.0005654 | nucleoplasm | ABL1,AKT1,ESR1,EZH2,FGFR2,HDAC1,HDAC4,HIF1A,JUN,MECP2,MEN1,MTOR,NR1I2,NR3C1,PDCD4,PPARA,PPARD,PPARG,PTEN,PTGES3,RORC,SGK1,SMAD2,SMAD4,STAT1,STAT3,TP53,VDR,VHL |
| GO.0005829 | cytosol | ABL1,AKT1,CCL3,CCR9,CYGB,ESR1,HDAC1,HDAC4,HIF1A,JUN,MECP2,MEN1,MTOR,NEDD8,NISCH,NR3C1,PAK1,PDCD4,PPARG,PTEN,PTGES3,RHOA,SGK1,SMAD2,SMAD4,STAT1,STAT3,TGM2,TP53,VHL |
| GO.0043233 | organelle lumen | ABL1,AKT1,EGFR,ESR1,EZH2,FGFR2,HDAC1,HDAC4,HIF1A,JUN,MECP2,MEN1,MTOR,NR1I2,NR3C1,NUAK1,PDCD4,PPARA,PPARD,PPARG,PTEN,PTGES3,RORC,SGK1,SMAD2,SMAD4,SMPD1,STAT1,STAT3,TF,TIMP1,TP53,VDR,VHL |
| GO.0044428 | nuclear part | ABL1,AKT1,EGFR,ESR1,EZH2,FGFR2,HDAC1,HDAC4,HIF1AJUN,MECP2,MEN1,MTOR,NR1I2,NR3C1,NUAK1,PAK1,PDCD4,PPARA,PPARD,PPARG,PTEN,PTGES3,RORC,SGK1,SMAD2,SMAD4,STAT1,STAT3,TP53,VDR,VHL |
| GO.0031981 | nuclear lumen | ABL1,AKT1,ESR1,EZH2,FGFR2,HDAC1,HDAC4,HIF1A,JUN,MECP2,MEN1,MTOR,NR1I2,NR3C1,NUAK1,PDCD4,PPARA,PPARD,PPARG,PTEN,PTGES3,RORC,SGK1,SMAD2,SMAD4,STAT1,STAT3,TP53,VDR,VHL |
| GO.0000785 | chromatin | BRD4,ESR1,EZH2,HDAC1,JUN,MECP2,MEN1,SMAD2,SMAD4,STAT1,STAT3,TP5 |
| GO.0070013 | intracellular organelle lumen | ABL1,AKT1,ESR1,EZH2,FGFR2,HDAC1,HDAC4,HIF1A,JUN,MECP2,MEN1,MTOR,NR1I2,NR3C1,NUAK1,PDCD4,PPARA,PPARD,PPARG,PTEN,PTGES3,RORC,SGK1,SMAD2,SMAD4,SMPD1,STAT1,STAT3,TP53,VDR,VHL |
| GO.0000790 | nuclear chromatin | BRD4,ESR1,EZH2,HDAC1,JUN,SMAD2,SMAD4,STAT1,STAT3,TP53 |
| GO.0044427 | chromosomal part | BRD4,ESR1,EZH2,HDAC1,JUN,MECP2,MEN1,PTGES3,SMAD2,SMAD4,STAT1,STAT3,TP53 |
| GO.0044422 | organelle part | ABL1,CPT1C,DPP4,EGFR,ESR1,EZH2,FGFR2,HBEGF,HDAC1,HDAC4,HIF1A,JUN,MECP2,MEN1,MMP2,MTOR,NR1I2,NR3C1,NUAK1,PAK1,PDCD4,PPARA,PPARD,PPARG,PTEN,PTGES3,RHOA,RORC,SGK1,SMAD2,SMAD4,SMPD1,STAT1,STAT3,STK25,TF,TIMP1,TP53,VDR,VHL |

**Table S6.** Main molecule function significantly relating to targets.

| #pathway ID | pathway description | targets |
| --- | --- | --- |
| GO.0005515 | protein binding | ABL1,ACE2,AKT1,BRD4,CCL2,CCL3,DPP4,  EGFR,ESR1,FGFR2,HBEGF,HDAC1,HDAC4,  HDAC7,HIF1A,IGF1R,IL17A,JUN,MECP2,MEN1,  MTOR,NEDD8,NISCH,NUAK1,PAK1,PPARA,  PPARG,PTEN,PTGES3,RHOA,SMAD2,SMAD4,  STAT1,STAT3,STK25,TF,TIMP1,TP53,VDR,VHL |
| GO.0004879 | RNA polymerase II transcription factor activity, ligand-activated sequence-specific DNA binding | ESR1,NR1I2,PPARA,PPARD,PPARG,RORC,  STAT3,VDR |
| GO.0003707 | steroid hormone receptor activity | ESR1,NR1I2,NR3C1,PPARA,PPARD,PPARG,  RORC,VDR |
| GO.0008134 | transcription factor binding | ESR1,HDAC1,HDAC4,HDAC7,HIF1A,JUN,  MTOR,PPARA,PPARG,SMAD2,STAT3,TP53,  VDR,VHL |
| GO.0043565 | sequence-specific DNA binding | ESR1,EZH2,HDAC1,HIF1A,JUN,MECP2,MTOR,  NR1I2,NR3C1,PPARA,PPARD,PPARG,RORC,  SMAD2,SMAD4,STAT1,STAT3,VDR |
| GO.0019899 | enzyme binding | ABL1,AKT1,DPP4,EGFR,ESR1,HDAC1,HDAC4,  HDAC7,HIF1A,JUN,NEDD8,PAK1,PPARA,PPARG,  PTEN,SMAD2,STAT1,STAT3,TIMP1,TP53,VHL |
| GO.0044212 | transcription regulatory region DNA binding | ESR1,EZH2,HDAC1,HDAC4,JUN,MEN1,MTOR,  NR1I2,NR3C1,PPARA,PPARG,SMAD2,SMAD4,  STAT1,STAT3,TP53 |
| GO.0000981 | RNA polymerase II transcription factor activity, sequence-specific DNA binding | ESR1,HIF1A,NR1I2,NR3C1,PPARA,PPARD,  PPARG,RORC,SMAD2,SMAD4,STAT1,STAT3,  TP53,VDR |
| GO.0003682 | chromatin binding | BRD4,EGFR,ESR1,EZH2,HDAC4,HDAC7,JUN,  MECP2,MEN1,PPARG,SMAD2,SMAD4,TP53 |
| GO.0001228 | transcriptional activator activity, RNA polymerase II transcription regulatory region sequence-specific binding | ESR1,HIF1A,JUN,NR1I2,NR3C1,PPARA,PPARG,  SMAD2,SMAD4,STAT3,TP53 |

**Table S7. Target proteins identified by network analysis.**

| No | Target | No | Target | No | Target | No | Target |
| --- | --- | --- | --- | --- | --- | --- | --- |
| 1 | ACE2 | 11 | FGFR2 | 21 | MMP2 | 31 | RAGE |
| 2 | AKT1 | 12 | HBEGF | 22 | MTOR | 32 | RHOA |
| 3 | ASM | 13 | HDAC1 | 23 | NEDD8 | 33 | SMAD2 |
| 4 | BRD4 | 14 | HDAC4 | 24 | NR1I2 | 34 | SMAD4 |
| 5 | CCL2 | 15 | HIF1A | 25 | P53 | 35 | STAT1 |
| 6 | CCL3 | 16 | IGF1R | 26 | PDCD4 | 36 | STAT3 |
| 7 | CYGB | 17 | IL17 | 27 | PPARA | 37 | TF |
| 8 | DPP4 | 18 | JUN | 28 | PPARD | 38 | TPO |
| 9 | EGFR | 19 | MECP2 | 29 | PPARG | 39 | VDR |
| 10 | EZH2 | 20 | MMP12 | 30 | PTEN | 40 | VHL |

**Table S8.** Main molecule function significantly relating to targets.

| No | Target name | Agonist/Antagonist | Effect | Reference |
| --- | --- | --- | --- | --- |
| 1 | PTEN | Antagonist | Reduction in type I collagen in hepatocytes | ([Yu et al., 2017](#_ENREF_22)) |
| 2 | PPARA | Agonist | Inhibition of the inflammatory response, and enhancement of SOD antioxidant activity | ([Xie et al., 2013](#_ENREF_18)) |
| 3 | RhoA | Antagonist | Inhibiting TGFβ1/CTGF pathway and α-SMA expression | ([Zhou et al., 2014](#_ENREF_23)) |
| 4 | SMAD2 | Antagonist | Preventing activation of the TGF-β/Smad signaling pathway | ([Liu et al., 2018](#_ENREF_9)) |
| 5 | SMAD4 | Antagonist | Suppressing profibrogenic effects of TGFβ1 | ([Zou et al., 2017](#_ENREF_24)) |
| 6 | STAT1 | Agonist | Induction of activated HSC apoptosis | ([Wu et al., 2011](#_ENREF_17)) |
| 7 | STAT3 | Antagonist | Promotes HCSs survival, proliferation, and activation | ([Nunez Lopez et al., 2016](#_ENREF_11)) |
| 8 | TGM2 | Antagonist | Lowered activation of Toll-like receptor 4 signaling and a reduced α-SMA expression | ([Wen et al., 2017](#_ENREF_15)) |
| 9 | VDR | Agonist | Downregulating the expression of type I collagen both α1 and α2 | ([Wan et al., 2016](#_ENREF_13)) |
| 10 | VHL | Antagonist | Inhibiting hepatic stellate cells activation and proliferation and promoted apoptosis | ([Wang et al., 2017](#_ENREF_14)) |
| 11 | ACE2 | Agonist | Recovering and rebuilding self-regulation of the RAS | ([Wu et al., 2015](#_ENREF_16)) |
| 12 | AKT1 | Antagonist | Suppressing the activation of HSCs | ([Yamaguchi et al., 2017](#_ENREF_19)) |
| 13 | CCR9 | Antagonist | Inhibiting the activation of HSCs | ([Chu et al., 2013](#_ENREF_3)) |
| 14 | EGFR | Antagonist | Mediating HSC activation and promoted a pro-fibrogenic phenotype | ([Liang et al., 2018](#_ENREF_8)) |
| 15 | HDAC1 | Antagonist | Inhibiting the activation of HSCs | ([Liu et al., 2013](#_ENREF_10)) |
| 16 | HIF1A | Antagonist | Preventing stimulus of epithelial to mesenchymal transition | ([Copple, 2010](#_ENREF_4)) |
| 17 | IGF1R | Antagonist | Inhibiting the TGF-β1-induced proliferation of HSCs | ([Yang et al., 2016](#_ENREF_20)) |
| 18 | MMP2 | Antagonist | Reducing the protein expression levels of α‑smooth muscle actin and type I collagen | ([Li et al., 2015](#_ENREF_7)) |
| 19 | mTOR | Antagonist | Inhibiting the activation of HSCs | ([Thiyagarajan et al., 2017](#_ENREF_12)) |
| 20 | NEDD8 | Antagonist | Reduction of HSCs activation | ([Zubiete-Franco et al., 2017](#_ENREF_25)) |
| 21 | CCL2 | Antagonist | Promoting monocyte/macrophage recruitment and tissue infiltration, as well as hepatic stellate cell activation | ([Lefebvre et al., 2016](#_ENREF_6)) |
| 22 | JUN | Antagonist | Decreasing platelet-derived growth factor and TGF-beta signaling in human HSCs | ([Kluwe et al., 2010](#_ENREF_5)) |
| 23 | FGFR2 | Agonist | Reducing expression of genes that control detoxification | ([Bohm et al., 2010](#_ENREF_2)) |
| 24 | PPARG | Agonist | Attenuating stellate cell activation | ([Yang et al., 2006](#_ENREF_21)) |
| 25 | SGK1 | Antagonist | downregulating the inflammatory transcription factor nuclear factor-κB, | ([Artunc and Lang, 2014](#_ENREF_1)) |

**Reference**

Artunc, F., and Lang, F. (2014). Mineralocorticoid and SGK1-sensitive inflammation and tissue

fibrosis. *Nephron Physiol* 128**,** 35-39. doi: 10.1159/000368267

Bohm, F., Speicher, T., Hellerbrand, C., Dickson, C., Partanen, J.M., Ornitz, D.M., and Werner, S.

(2010). FGF receptors 1 and 2 control chemically induced injury and compound detoxification in

regenerating livers of mice. *Gastroenterology* 139**,** 1385-1396. doi: 10.1053/j.gastro.2010.06.069

Chu, P.S., Nakamoto, N., Ebinuma, H., Usui, S., Saeki, K., Matsumoto, A., Mikami, Y., Sugiyama, K., Tomita, K., Kanai, T., Saito, H., and Hibi, T. (2013). C-C motif chemokine receptor 9 positive macrophages activate hepatic stellate cells and promote liver fibrosis in mice. *Hepatology* 58**,** 337-350. doi: 10.1002/hep.26351

Copple, B.L. (2010). Hypoxia stimulates hepatocyte epithelial to mesenchymal transition by hypoxia-inducible factor and transforming growth factor-beta-dependent mechanisms. *Liver Int* 30**,** 669-682. doi: 10.1111/j.1478-3231.2010.02205.x

Kluwe, J., Pradere, J.P., Gwak, G.Y., Mencin, A., De Minicis, S., Osterreicher, C.H., Colmenero, J., Bataller, R., and Schwabe, R.F. (2010). Modulation of hepatic fibrosis by c-Jun-N-terminal kinase inhibition. *Gastroenterology* 138**,** 347-359. doi: 10.1053/j.gastro.2009.09.015

Lefebvre, E., Moyle, G., Reshef, R., Richman, L.P., Thompson, M., Hong, F., Chou, H.L., Hashiguchi, T., Plato, C., Poulin, D., Richards, T., Yoneyama, H., Jenkins, H., Wolfgang, G., and Friedman, S.L. (2016). Antifibrotic Effects of the Dual CCR2/CCR5 Antagonist Cenicriviroc in Animal Models of Liver and Kidney Fibrosis. *PLoS One* 11**,** e0158156. doi: 10.1371/journal.pone.0158156

Li, Y., Liu, F., Ding, F., Chen, P., and Tang, M. (2015). Inhibition of liver fibrosis using vitamin A-coupled liposomes to deliver matrix metalloproteinase-2 siRNA in vitro. *Mol Med Rep* 12**,** 3453-3461. doi: 10.3892/mmr.2015.3842

Liang, D., Chen, H., Zhao, L., Zhang, W., Hu, J., Liu, Z., Zhong, P., Wang, W., Wang, J., and Liang, G. (2018). Inhibition of EGFR attenuates fibrosis and stellate cell activation in diet-induced model of nonalcoholic fatty liver disease. *Biochim Biophys Acta* 1864**,** 133-142. doi: 10.1016/j.bbadis.2017.10.016

Liu, H., Dong, F., Li, G., Niu, M., Zhang, C., Han, Y., He, L., Yin, P., Wang, B., Sang, X., Li, R., Wang, J., Bai, Z., and Xiao, X. (2018). Liuweiwuling tablets attenuate BDL-induced hepatic fibrosis via modulation of TGF-beta/Smad and NF-kappaB signaling pathways. *J Ethnopharmacol* 210**,** 232-241. doi: 10.1016/j.jep.2017.08.029

Liu, Y., Wang, Z., Wang, J., Lam, W., Kwong, S., Li, F., Friedman, S.L., Zhou, S., Ren, Q., Xu, Z., Wang, X., Ji, L., Tang, S., Zhang, H., Lui, E.L., and Ye, T. (2013). A histone deacetylase inhibitor, largazole, decreases liver fibrosis and angiogenesis by inhibiting transforming growth factor-beta and vascular endothelial growth factor signalling. *Liver Int* 33**,** 504-515. doi: 10.1111/liv.12034

Nunez Lopez, O., Bohanon, F.J., Wang, X., Ye, N., Corsello, T., Rojas-Khalil, Y., Chen, H., Chen, H., Zhou, J., and Radhakrishnan, R.S. (2016). STAT3 Inhibition Suppresses Hepatic Stellate Cell Fibrogenesis: HJC0123, a Potential Therapeutic Agent for Liver Fibrosis. *RSC Adv* 6**,** 100652-100663. doi: 10.1039/c6ra17459k

Thiyagarajan, V., Lee, K.W., Leong, M.K., and Weng, C.F. (2017). Potential natural mTOR inhibitors screened by in silico approach and suppress hepatic stellate cells activation. *J Biomol Struct Dyn***,** 1-15. doi: 10.1080/07391102.2017.1411295

Wan, L.Y., Zhang, Y.Q., Li, J.M., Tang, H.Q., Chen, M.D., Ni, Y.R., Huang, H., Liu, C.B., and Wu, J.F. (2016). Liganded Vitamin D Receptor Through Its Interacting Repressor Inhibits the Expression of Type I Collagen alpha1. *DNA Cell Biol* 35**,** 498-505. doi: 10.1089/dna.2016.3367

Wang, J., Lu, Z., Xu, Z., Tian, P., Miao, H., Pan, S., Song, R., Sun, X., Zhao, B., Wang, D., Ma, Y., Song, X., Zhang, S., Liu, L., and Jiang, H. (2017). Reduction of hepatic fibrosis by overexpression of von Hippel-Lindau protein in experimental models of chronic liver disease. *Sci Rep* 7**,** 41038. doi: 10.1038/srep41038

Wen, Z., Ji, X., Tang, J., Lin, G., Xiao, L., Liang, C., Wang, M., Su, F., Ferrandon, D., and Li, Z. (2017). Positive Feedback Regulation between Transglutaminase 2 and Toll-Like Receptor 4 Signaling in Hepatic Stellate Cells Correlates with Liver Fibrosis Post Schistosoma japonicum Infection. *Front Immunol* 8**,** 1808. doi: 10.3389/fimmu.2017.01808

Wu, L., Zhou, P.Q., Xie, J.W., Zhu, R., Zhou, S.C., Wang, G., Wu, Z.X., and Hao, S. (2015). Effects of Yinchenhao decoction on self-regulation of renin-angiotensin system by targeting angiotensin converting enzyme 2 in bile duct-ligated rat liver. *J Huazhong Univ Sci Technolog Med Sci* 35**,** 519-524. doi: 10.1007/s11596-015-1463-9

Wu, X.X., Wu, L.M., Fan, J.J., Qin, Y., Chen, G., Wu, X.F., Shen, Y., Sun, Y., and Xu, Q. (2011). Cortex Dictamni extract induces apoptosis of activated hepatic stellate cells via STAT1 and attenuates liver fibrosis in mice. *J Ethnopharmacol* 135**,** 173-178. doi: 10.1016/j.jep.2011.03.010

Xie, C., Li, L., Xu, Y.P., Zhu, Y.Y., and Jiang, J.J. (2013). [Anti-fibrosis effects of fenofibrate in mice with hepatic fibrosis]. *Zhonghua Gan Zang Bing Za Zhi* 21**,** 914-919.

Yamaguchi, M., Saito, S.Y., Nishiyama, R., Nakamura, M., Todoroki, K., Toyo'oka, T., and Ishikawa, T. (2017). Caffeine Suppresses the Activation of Hepatic Stellate Cells cAMP-Independently by Antagonizing Adenosine Receptors. *Biol Pharm Bull* 40**,** 658-664. doi: 10.1248/bpb.b16-00947

Yang, J.J., Liu, L.P., Tao, H., Hu, W., Shi, P., Deng, Z.Y., and Li, J. (2016). MeCP2 silencing of LncRNA H19 controls hepatic stellate cell proliferation by targeting IGF1R. *Toxicology* 359-360**,** 39-46. doi: 10.1016/j.tox.2016.06.016

Yang, L., Chan, C.C., Kwon, O.S., Liu, S., Mcghee, J., Stimpson, S.A., Chen, L.Z., Harrington, W.W., Symonds, W.T., and Rockey, D.C. (2006). Regulation of peroxisome proliferator-activated receptor-gamma in liver fibrosis. *Am J Physiol Gastrointest Liver Physiol* 291**,** G902-911. doi: 10.1152/ajpgi.00124.2006

Yu, F., Chen, B., Dong, P., and Zheng, J. (2017). HOTAIR Epigenetically Modulates PTEN Expression via MicroRNA-29b: A Novel Mechanism in Regulation of Liver Fibrosis. *Mol Ther* 25**,** 205-217. doi: 10.1016/j.ymthe.2016.10.015

Zhou, H., Fang, C., Zhang, L., Deng, Y., Wang, M., and Meng, F. (2014). Fasudil hydrochloride hydrate, a Rho-kinase inhibitor, ameliorates hepatic fibrosis in rats with type 2 diabetes. *Chin Med J (Engl)* 127**,** 225-231.

Zou, Y., Cai, Y., Lu, D., Zhou, Y., Yao, Q., and Zhang, S. (2017). MicroRNA-146a-5p attenuates liver fibrosis by suppressing profibrogenic effects of TGFbeta1 and lipopolysaccharide. *Cell Signal* 39**,** 1-8. doi: 10.1016/j.cellsig.2017.07.016

Zubiete-Franco, I., Fernandez-Tussy, P., Barbier-Torres, L., Simon, J., Fernandez-Ramos, D., Lopitz-Otsoa, F., Gutierrez-De Juan, V., De Davalillo, S.L., Duce, A.M., Iruzubieta, P., Taibo, D., Crespo, J., Caballeria, J., Villa, E., Aurrekoetxea, I., Aspichueta, P., Varela-Rey, M., Lu, S.C., Mato, J.M., Beraza, N., Delgado, T.C., and Martinez-Chantar, M.L. (2017). Deregulated neddylation in liver fibrosis. *Hepatology* 65**,** 694-709. doi: 10.1002/hep.28933
